# Effectiveness of bio-effectors on maize, wheat and tomato performance and phosphorus acquisition from greenhouse to field scales in Europe and Israel: a meta-analysis

**DOI:** 10.3389/fpls.2024.1333249

**Published:** 2024-04-02

**Authors:** Peteh Mehdi Nkebiwe, Jonas D. Stevens Lekfeldt, Sarah Symanczik, Cécile Thonar, Paul Mäder, Asher Bar-Tal, Moshe Halpern, Borbala Biró, Klára Bradáčová, Pedro C. Caniullan, Krishna K. Choudhary, Vincenza Cozzolino, Emilio Di Stasio, Stefan Dobczinski, Joerg Geistlinger, Angelika Lüthi, Beatriz Gómez-Muñoz, Ellen Kandeler, Flora Kolberg, Zsolt Kotroczó, Martin Kulhanek, Filip Mercl, Guy Tamir, Narges Moradtalab, Alessandro Piccolo, Albino Maggio, Dinah Nassal, Magdolna Zita Szalai, Katalin Juhos, Ciprian G. Fora, Andreea Florea, Gheorghe Poşta, Karl Fritz Lauer, Brigitta Toth, Pavel Tlustoš, Isaac K. Mpanga, Nino Weber, Markus Weinmann, Uri Yermiyahu, Jakob Magid, Torsten Müller, Günter Neumann, Uwe Ludewig, Andreas de Neergaard

**Affiliations:** ^1^ Institute of Crop Science, Departments of Nutritional Crop Physiology and Fertilization and Soil Matter Dynamics, University of Hohenheim, Stuttgart, Germany; ^2^ Faculty of Science, Department of Plant and Environmental Sciences, University of Copenhagen, Frederiksberg C, Denmark; ^3^ Department of Soil Sciences, Research Institute of Organic Agriculture FiBL, Frick, Switzerland; ^4^ Institute of Plant Sciences, Agricultural Research Organization (ARO), Rishon LeZion, Israel; ^5^ Gilat Research Center, Agricultural Research Organization, Gilat, Israel; ^6^ Department of Agro-Environmental Studies, Hungarian University of Agriculture and Life Sciences, Budapest, Hungary; ^7^ Centro Interdipartimentale di Ricerca sulla Risonanza Magnetica Nucleare per l’Ambiente, l’Agro-Alimentare ed i Nuovi Materiali (CERMANU), Università di Napoli Federico II, Portici, Italy; ^8^ Department of Agricultural Sciences, University of Napoli Federico II, Portici, Italy; ^9^ Institute of Bioanalytical Sciences, Anhalt University of Applied Sciences, Bernburg, Germany; ^10^ Institute of Soil Science and Land Evaluation, Soil Biology Department, University of Hohenheim, Stuttgart, Germany; ^11^ Department of Agro-Environmental Chemistry and Plant Nutrition, Czech University of Life Sciences in Prague, Suchdol, Czechia; ^12^ Department of Horticulture, Banat’s University of Agricultural Sciences and Veterinary Medicine “King Michael I of Romania”, Timișoara, Romania; ^13^ Institute of Food Science, Faculty of Agricultural and Food Sciences and Agricultural Management, University of Debrecen, Debrecen, Hungary; ^14^ Roskilde University, Roskilde, Denmark

**Keywords:** meta-analysis, PGPMs, biostimulants, biofertilizers, phosphorus, maize, wheat, tomato

## Abstract

Biostimulants (Bio-effectors, BEs) comprise plant growth-promoting microorganisms and active natural substances that promote plant nutrient-acquisition, stress resilience, growth, crop quality and yield. Unfortunately, the effectiveness of BEs, particularly under field conditions, appears highly variable and poorly quantified. Using random model meta-analyses tools, we summarize the effects of 107 BE treatments on the performance of major crops, mainly conducted within the EU-funded project BIOFECTOR with a focus on phosphorus (P) nutrition, over five years. Our analyses comprised 94 controlled pot and 47 field experiments under different geoclimatic conditions, with variable stress levels across European countries and Israel. The results show an average growth/yield increase by 9.3% (n=945), with substantial differences between crops (tomato > maize > wheat) and growth conditions (controlled nursery + field (Seed germination and nursery under controlled conditions and young plants transplanted to the field) > controlled > field). Average crop growth responses were independent of BE type, P fertilizer type, soil pH and plant-available soil P (water-P, Olsen-P or Calcium acetate lactate-P). BE effectiveness profited from manure and other organic fertilizers, increasing soil pH and presence of abiotic stresses (cold, drought/heat or salinity). Systematic meta-studies based on published literature commonly face the inherent problem of publication bias where the most suspected form is the selective publication of statistically significant results. In this meta-analysis, however, the results obtained from all experiments within the project are included. Therefore, it is free of publication bias. In contrast to reviews of published literature, our unique study design is based on a common standardized protocol which applies to all experiments conducted within the project to reduce sources of variability. Based on data of crop growth, yield and P acquisition, we conclude that application of BEs can save fertilizer resources in the future, but the efficiency of BE application depends on cropping systems and environments.

## 1 Introduction

Over the past century, improvements in agricultural productivity have mainly been driven by the introduction of high-yielding crop varieties combined with the intensive use of agrochemicals (Tilman et al., 2002). However, excessive use of nitrogen (N), phosphate (P) fertilizers, and pesticides has created an array of environmental problems such as groundwater pollution, eutrophication of surface waters, and increased emissions of ammonia (NH_3_) and nitrous oxide (N_2_O) (Tilman et al., 2002). For P fertilizers, the large mining efforts for rock phosphate precursors and the high rates of P fertilization carried out during the last century led to a “high risk” perturbation of the P cycle (Steffen et al., 2015). At the same time, rock phosphate is a finite resource and high-quality reserves with low co-contamination by toxic heavy metals, are concentrated in a few places around the world (Cordell et al., 2009). Due to historical surpluses in P inputs, large quantities of P have accumulated in most agricultural soils in Europe (Withers et al., 2015). However, 99% of the total P in soil is present in P fractions with strongly limited availability for root uptake, which requires the presence of phosphate anions in the soil solution (Zou et al., 1992; Richardson, 2001). Major soil P fractions comprise inorganic P (P_i_) and organic P (P_o_) sequestered in soil organic matter (SOM). P_i_ may be adsorbed to mineral surfaces with Fe/Al oxides and hydroxides, precipitated with calcium (Ca), aluminum (Al), and iron (Fe) or adsorbed to SOM (Hinsinger, 2001; Richardson and Simpson, 2011). Furthermore, a large proportion of soluble P added with fertilizers rapidly becomes unavailable via fixation and will no longer be directly available for plant uptake. Nutrient acquisition can be further impaired by stress factors affecting root development, with increasing impact related to climate change.

Strategies for decreasing the input of N and P fertilizers in agroecosystems and enhancing nutrient use efficiencies include the use of fertilizers based on products of waste recycling (Möller et al., 2018), appropriate timing and placement of fertilizers (Dunbabin et al., 2009; Nkebiwe et al., 2016a), crop genetic potential (van de Wiel et al., 2016) and bio-effectors (BEs) with plant growth-promoting properties (Herrmann et al., 2022). BEs lack significant amounts of nutrients and include a diverse group of living microorganisms and active natural compounds (Weinmann, 2017). To evaluate the potential of BE-assisted production strategies, the integrated project BIOFECTOR (www.biofector.info; located within the EU 7th framework program) was initiated in 2012 with the aim to investigate perspectives for reducing the input of mineral fertilizers (especially P) and to improve stress resilience in European crop production. The BEs tested included viable plant growth-promoting microorganisms (PGPMs), natural active substances based on extracts from seaweed, plants or compost preparations, humic acids, as well as amino acids, protein- or chitin-hydrolysates (Backer et al., 2018; Halpern et al., 2015).

The term “bio-effector” was coined to cover the whole range of plant growth-promoting properties by microorganisms (PGPMs) and natural active substances (non-microbial biostimulants). The separation of plant growth-promoting properties into categories of bio-control agents acting against pests and pathogens and bio-stimulants with other beneficial functions was intentionally avoided. A whole suite of different mechanisms may be responsible for the plant growth-promoting effect of BEs (de et al., 2015), acting directly or via interactions with native soil organisms. Common modes of action of both microbial and non-microbial BEs, are the induction of plant defense mechanisms against abiotic and biotic stress factors via elicitor-based signaling events (Backer et al., 2018; Thoms et al., 2021) and the stimulation of root growth via direct or indirect interactions with plant hormonal balances (Richardson, 2001; Mäder et al., 2011; Richardson et al., 2011; Bradáčová et al., 2019b; Mpanga et al., 2019b; Moradtalab et al., 2020). Adaptive changes in root morphology are particularly important for the absorption of nutrients with low solubility and mobility in soils such as P (Vacheron et al., 2013). Shifts in the plant hormonal balance can alter root branching, fine root production and root hair development and thus improve plant nutrient acquisition not only due to an increased root surface (Richardson, 2001; Mäder et al., 2011), but also through increased root exudation (Richardson et al., 2011).

Plant-available soil nutrients are an important determinant of the function of BEs (Leggett et al., 2015; Egamberdiyeva, 2007) and the combined application of fertilizers (mineral or organic) with BEs may increase nutrient availability (Gómez-Muñoz et al., 2017; Nkebiwe et al., 2017). Soil pH (Sánchez-Esteva et al., 2016), SOM (Schütz et al., 2018) and the size, composition and activity of the native soil microbial community (Mäder et al., 2011) are important. Wide differences of the effects of BEs on the performance of different plant species and cultivars (Marasco et al., 2013; Timmusk et al., 2014), BE source and application rate (Rose et al., 2014) and across geoclimatic regions (Rose et al., 2014) are observed. Combinations of different strains of PGPMs or non-microbial BEs with complementary and synergistic properties may lead to a larger effect than application of single BEs (Omar, 1997; Han and Lee, 2006; Borriss, 2015; Barea et al., 2005; Bona et al., 2017). BEs may improve plant tolerance to abiotic and biotic stresses (van Oosten et al., 2017).

A steadily increasing number of reviews and meta-analyses on different types of BEs suggests effectiveness of *Azospirillum* spp (Veresoglou and Menexes, 2010)., plant growth-promoting rhizobacteria (PGPR) (Rubin et al., 2017) as well as other microbial and non-microbials BEs such as humic substances (Herrmann et al., 2022; Rose et al., 2014). But there is a large variation in the effects observed after BE application (Schütz et al., 2018; Rose et al., 2014). This may be because systematic meta-studies based on published literature commonly face the inherent problem of *publication bias* where the most suspected form is the selective publication of statistically significant results (Rosenberg, 2005). Furthermore, it has often been reported that effects observed in pot experiments under controlled conditions could not be translated to the field (Richardson and Simpson, 2011). Part of the challenge lies in the fact that the mechanisms behind the observed positive effects are often not known (Yakhin et al., 2017). Therefore, in our contribution to close these knowledge gaps, we have conducted a meta-analysis in which the results obtained from all experiments within the BIOFECTOR project are included. Therefore, it is free of *publication bias*. Moreover. In contrast to reviews of published literature, our unique study design is based on a common standardized protocol which applies to all experiments conducted within the project to reduce sources of variability. The overall hypothesis of the study was that environmental conditions can be identified that favor BE effectiveness. Special emphasis was placed on P as a critical macronutrient for the following reasons: a) It has limited plant availability; b) BEs can induce physical, chemical and biological modifications in plant roots and rhizosphere to favor adaptation to P limitation, which may be beneficial for the acquisition of other nutrients (e.g. stimulation of root growth, rhizosphere acidification, promotion of mycorrhizal associations). Using a common experimental protocol, experiments were primarily conducted with three important crop species representing the European crop production systems: maize (*Zea mays L*.); wheat (*Triticum aestivum L*.) and tomato (*Solanum lycopersicum L.).* Furthermore, a wide range of BE treatments, soils and fertilizers were used in different locations and climatic conditions across Europe and Israel. Data were produced during the years 2013 – 2017 and mean effects of BE application were quantified. Using moderator analysis, we identified experimental conditions under which positive BE effects are most likely to be observed.

## 2 Materials and methods

### 2.1 Data source

**Figure 1:**
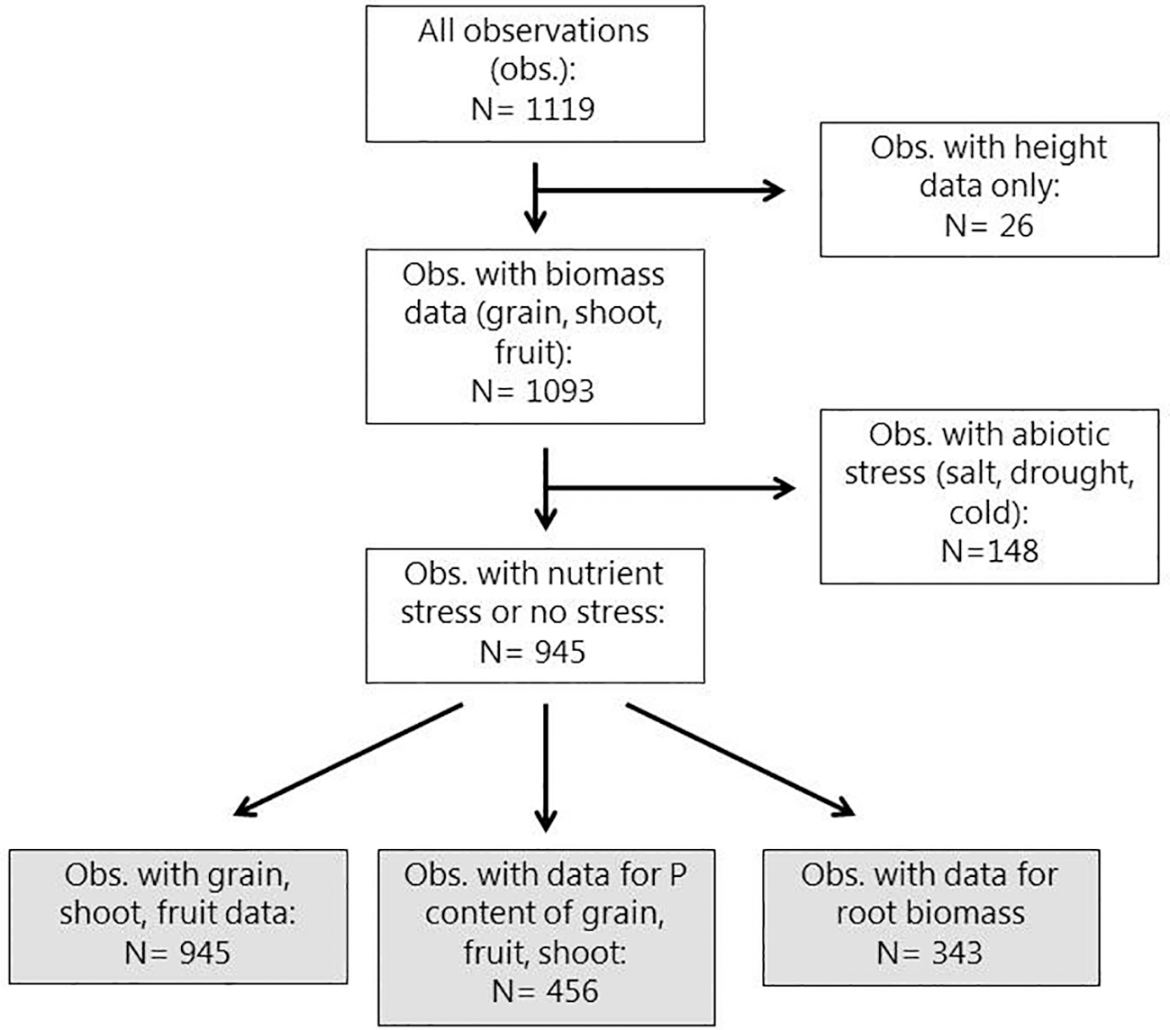
Diagram showing the flow of observations in initial handling of data for the meta-analyses. The grey boxes represent subsets of observations used for meta-analyses in the present paper. After excluding the 26 datasets containing observations of plant height only, the remaining 1093 datasets were produced from 136 experiments (47 field and 89 pot) by 16 project partners across the European Union and Israel from 2013 – 2017.

Experiments were conducted during the years 2013-2017 by 16 BioFector partner institutions (**Supplementary Table S1**, Supplementary materials). Experimental data were collected directly from the doctoral students and staff responsible for conducting the experiments. This setup enabled us to obtain a considerable amount of background information on the experiments and to cross-check data inputs. Data were entered in a database made in Microsoft Excel. A description of the structure of the database is included in the supplementary information (**Supplementary Figure S1**). A total of 141 experiments (94 pot and 47 field) were performed. For field trials, an experiment was defined as a one-year growing season. So, if the same experiment was carried out during more than one year, the results from the different growing seasons were regarded as separate experiments. The eligibility criteria for inclusion in the meta-analysis were: (i) experiments had to include both treatments with addition of BEs and a corresponding control where all conditions were identical except that no BE was added (negative control for BE addition), (ii) data on at least one of the following yield variables must have been reported: shoot dry matter (DM), fruit DM, fruit fresh matter (FM) or grain DM, (iii) one of the three model crops (maize, tomato or wheat) were included. This led to the exclusion of observations from experiments that for instance only reported data on plant height and not DM (**Figure 1**). From 141 experiments, 136 experiments (89 pot and 47 field) met the eligibility criteria. These 136 experiments yielded 945 observations. An observation is defined as a unique control (untreated)-to-BE (treated) data pair. For each of these observations the number of replicates of the control and BE treatment were recorded and for any response variable (shoot biomass, grain yield etc.), the mean and standard deviation of the control and BE treatment were also recorded.

### 2.2 BE treatments

A large variety of different BE treatments were applied in the BioFector project (**Supplementary Table S2**, Supplementary materials). Here BE treatments included both experimental strains/formulations and already marketed products and combinations of single strains and/or extracts. The treatments were grouped according to four overall BE categories (**Table 1**): Single strain of bacteria; Single strain of fungus; Mixture; and Non-microbial. Investigations on arbuscular mycorrhizal Fungi (AMF) was not within the scope of the BIOFECTOR project. For this reason, there were no experiments conducted explicitly to investigate for AMF effects on crop performance, although AMF effects on root colonization by native AMF fungi were considered in some studies.

**Table 1:** Overview of BE categories used in this study.

Name of BE* category	Number of datasets	Examples of contents	Type BEs selected in BIOFECTOR	
			*Examples Organism/origin*	Product names
**Single strains of bacteria**	471	Isolates of soil bacteria (PGPR)	*Pseudomonas, Bacillus, Paenibacillus, Azotobacter*, etc.	Proradix, Rhizovital,
				Rhizovital42, ABiTEP
**Single strain of fungi**	163	Isolates of soil fungi	*Trichoderma*, Penecillium,	Trianum-P, Koppert
**Mixture**	183	> 1 strain PGPM + non-microbial´ BMs + Si, Zn, Mn	*T. harzianum* + *Bacillus* strains + Mn/Zn	Combifector A, AUAS
**Non-microbial**	128	Humic acids artichoke	N/A	N/A
		Extracts of seaweeds of the genera *Ascophyllum, Laminaria*	*Ascophyllum nodosum*	SuperFifty, BioAtlantis
		Extract of Sorghum roots, killed bacteria	N/A	N/A
	Total 945**			

*There were 139 different BEs of which 106 were tested in experiments included in the meta-analysis database.

**These 945 datasets exclude 148 datasets from experiments conducted under abiotic stress conditions (salinity, drought and cold). Put together, 1093 datasets were obtained from 136 experiments (47 field and 89 pot) by 16 project partners across the European Union and Israel from 2013 - 2017.

### 2.3 Crops

Three crops with importance for European agriculture were selected in the project representing C3 (wheat, *Triticum aestivum L*.) and C4 (maize, *Zea mays L*.) grain crops as well as fruiting crops (tomato, *Solanum lycopersicum L.)*. Maize was specifically selected due to its early sensitivity to P limitation (Colomb et al., 2000). A full list of cultivars used in the experiments is included as supplementary material (**Supplementary Table S3**, Supplementary materials). A comprehensive list of fertilizers applied is also given on **Supplementary Table S4** under supplementary materials.

### 2.4 Soil data

Soil characteristics (**Supplementary Table S5**) were generally obtained on air-dried soils analyzed at the *Landesanstalt für Landwirtschaftliche Chemie* (now renamed Core Facility) at the University of Hohenheim, Stuttgart, Germany. The standard methods of the *Verband Deutscher Landwirtschaftlicher Untersuchungs- und Forschungsanstalten* (VDLUFA) were used to analyze soil texture, organic carbon content and pH. Soil texture was analyzed according to the VDLUFA standard method C 2.2.1 (Vdlufa-Methodenbuch, 1991); soil organic carbon (SOC) content according to the VDLUFA standard method A 4.1.3.1 (Vdlufa-Methodenbuch, 1991); and soil pH in 0.01 M CaCl_2_ according to the VDLUFA standard method A 5.1.1 (Vdlufa-Methodenbuch, 1991). Finally, the plant-available soil P was measured using the calcium-acetate lactate-extractable P (P_CAL_) according to the VDLUFA standard method A 6.2.1.1 (Vdlufa-Methodenbuch, 1991), the Olsen-P (P_Olsen_) method (Olsen, 1954) or by water extraction method (P_water_) (Sissingh, 1971). Unlike residual soil P (P_resid_), which can only be extracted only by strong acids (e.g. HCl and H_2_SO_4_), P_CAL_, P_Olsen_ and P_water_, represent soil P fractions that are apparently available to plant roots and taken up. When data were available for more than one method, P_CAL_ was chosen if the pH was below 7.5 and P_Olsen_ was chosen if the pH was 7.5 or above. P_water_ was only chosen if data was not recorded using any of the two other methods. In pot experiments sand was added in most of the experiments (**Supplementary Table S1**) to ensure good substrate drainage in the pots. Therefore, the level of available P in the pot experiments was corrected for the addition of sand by assuming a simple dilution effect according to Equation 1:


(1) $$P_{growth\;medium}\;=P_{soil}\;\times \left(\frac{100\%-\%\;sand\;added}{100\%}\right)$$

The same calculation was performed for SOC content.

### 2.5 Response variables

Meta-analyses were conducted on the following response variables: (i) mass of grain, fruit or shoot, (ii) total P content of grain, fruit or shoot; (iii) root mass. In some experiments more than one yield parameter was measured (for instance straw biomass and grain yield). In these cases, one of the yield types was chosen for each experiment using the following precedence: grain>fruit>shoot biomass.

### 2.6 Meta-analyses

The response ratio was used as the effect size (Hedges et al., 1999). For each observation, the response ratio (RR) was calculated for the response variable in question according to Equation 2:


(2) $$RR=\frac{{\bar{X}}_{BE}}{{\bar{X}}_{control}}$$

where and ${\bar{X}}_{control}$ are the means of the BE treatment and the corresponding control treatment, respectively. This number is log-transformed according to Equation 3 to maintain symmetry in the analysis (Olkin et al., 2009):


(3) $$\ln\left(RR\right)=\ln\left(\frac{{\bar{X}}_{BE}}{{\bar{X}}_{control}}\right)=\ln\left({\bar{X}}_{BE}\right)-ln\left({\bar{X}}_{control}\right)$$

Calculations of effect size and variance of the individual observations were carried out with the escalc() function of the metafor package for R (Viechtbauer, 2010). Observation, cluster and experiment were included as random factors in multi-level model meta-analysis using the rma.mv() function of the metafor package. Either random (for main effects) or mixed-effects (for moderator analyses) meta-analyses were carried out using the restricted maximum likelihood (REML) estimator. A basic assumption when conducting meta-analysis is independence of data (Borenstein et al., 2011). However, in multiple treatment studies that all refer to one common control the effect sizes will be correlated (Olkin et al., 2009). Since all the experiments included in our study contributed with more than one observation in the analysis, these observations will therefore not all be independent, thus violating the assumption of independence. This may be handled by aggregating data within experiments (Gattinger et al., 2012), which is often advised (Del Re, 2015) but then information is lost in the analysis. An alternative would be to ignore the dependence of observations in the analyses, which has also been practiced (Schütz et al., 2018; Gattinger et al., 2012; Lori et al., 2017; Skinner et al., 2014). However, we took the dependence of effect size estimates with shared controls into account by not only including the variance of the individual effect size estimates but also the covariances of the dependent effect size estimates (belonging to the same control group). The covariance for each cluster of observations was calculated using data from the shared control according to Lajeunesse et al (Lajeunesse, 2011):


(4) $$covariance=\frac{{\left(sd_{control}\right)}^{2}}{N_{control}\cdot {\bar{X}}_{control}^{2}}$$

A variance-covariance matrix was then constructed with the variance estimates from escalc() and the covariances calculated using Equation 4. The resulting variance-covariance matrix was then used as argument in the rma.mv() function.

After analyzing overall BE effects, moderator (subgroup) analyses were carried out using the following moderators: crop type (maize, tomato, wheat); growing conditions (A) (for all crops: controlled, controlled nursery + field (Seed germination and nursery under controlled conditions and young plants transplanted to the field) and field); and growing conditions (B) (for maize only: controlled, field); BE type (four levels: single bacterium, single fungus, mixtures, microbial and non-microbial); fertilizer type based on different P forms and fertilizers based on products of waste recycling: (Control-no P fertilizer, ashes, biochar, compost, digestates, animal waste products, Rock P, sewage sludge, soluble P); soil pH (four levels:<5.5, 5.5-6.5, 6.5-7.5, 7.5-8.5; substrate plant-available P (three levels: low, moderate, optimal, high) and substrate concentration (% OC, five levels: 0-0.5, 0.5-1.0, 1.0-1.5, 1.5-2.0, 2.0-3.0); type of N fertilizer (three levels: organic N, other mineral N, stabilized ammonium); N or P fertilizer application method (four levels: No fertilizer, Fertigation, Placement, Broadcast). For plant-available soil P, we only looked at observations that originated from plots/pots that were not amended with a P fertilizer because the addition of P fertilizers is expected to influence the level of plant-available P in the soil. The models in most cases generated residuals, which were non-normally distributed. Although this is a violation of the assumptions behind the models, but the works of Kontopantelis & Reeves (Kontopantelis and Reeves, 2012; Kontopantelis and Reeves, 2010) indicates that this does not have the potential to fundamentally alter the conclusions. To avoid any selection biases that may occur in our case by rejecting datapoints considered as influential outliers (Habeck and Schultz, 2015), all datapoints were included in the meta-analysis as long as the criteria for experiments in that particular analysis were met.

## 3 Results

### 3.1 Geographic distribution of trials

**Figure 2:**
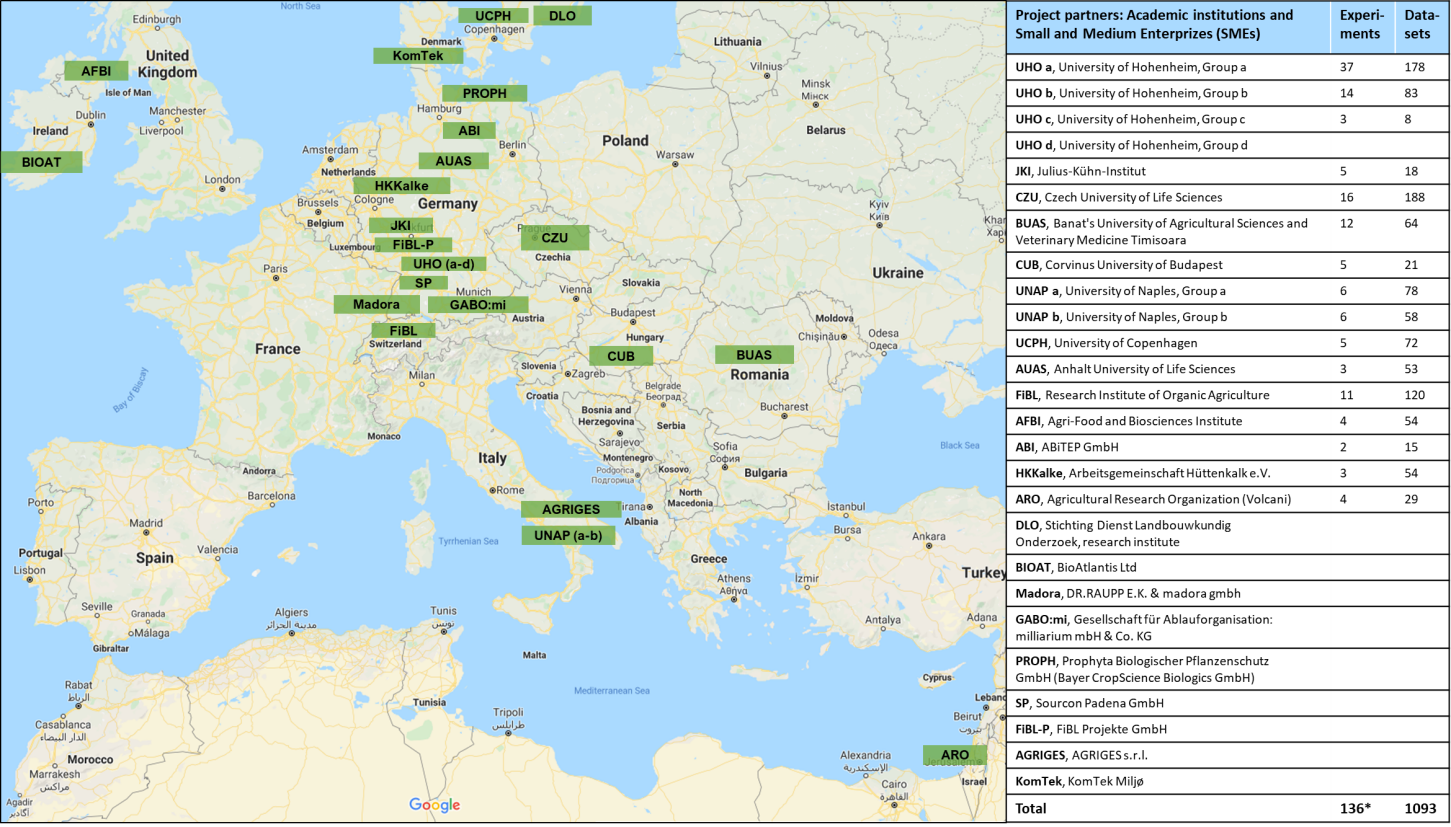
Geographical distribution of 16 project partners who provided 1093 datasets from 136 experiments across the European Union and Israel during the period 2013 – 2017.*136 experiments after excluding 5 experiments containing only plant height data from 141 experiments in total.

The location of BioFector project partner institutions across Europe and Israel is shown in **Figure 2** together with the number of experiments conducted and the resulting number of datasets or observations (BE versus control comparisons) provided by each partner. A total of 141 experiments were conducted from 2013 – 2017 (94 pot and 47 field) leading to 1119 observations (**Figure 1**). Excluding experiments where only plant height was recorded led to 1093 observations, originating from 136 experiments (89 pot and 47 field) (**Figures 1**, **2**). Out of these, 148 observations with abiotic stresses (cold, drought or salt) other than nutrient (P) limitation were pooled aside for separate analysis.

### 3.2 BE effects in the context of nutrient acquisition

**Figure 3:**
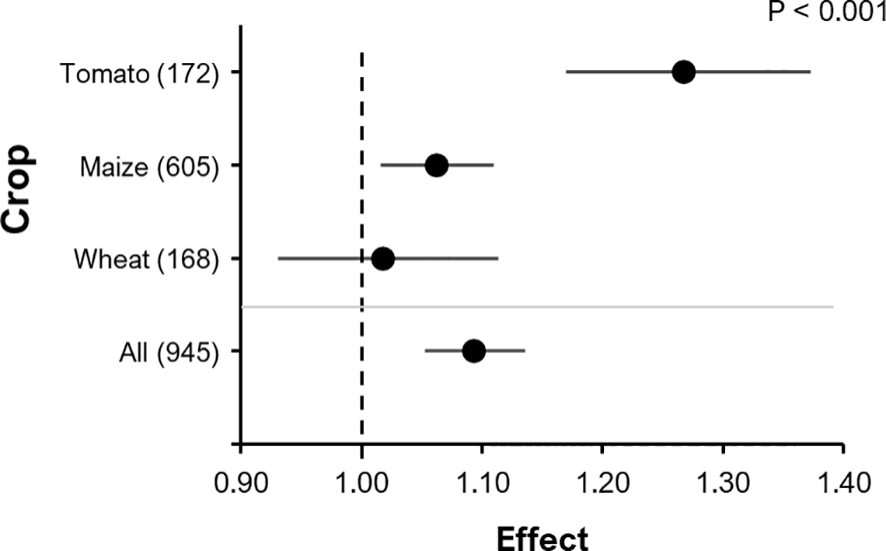
The effect of BE addition on either: grain DM, fruit DM, fruit FM or shoot DM according to different crops within all experiments. A total of 945 observations from 290 clusters were included in the analysis. For each crop, the number inside the brackets represent the number of observations included, the point indicates the mean effect while the horizontal line represents the 95% C.I. The p-value indicates whether there was a significant effect of crop type.

The RR for yield (e.g. shoot biomass, grain, fruit) from 945 observations of 73 pot and 41 field experiments that were ordered in 290 clusters was 1.093 (P< 0.0001; 95% C.I.: 1.053-1.135) (**Figure 3**). Observations within the same cluster were not independent (see details in Methods), as a cluster was defined as a group that shares a common control (Olkin et al., 2009). The RR of P content in grain, fruit or shoot from 456 observations belonging to 168 clusters and 53 experiments was 1.083 (P<0.001, 95% C.I.: 1.037-1.131). Furthermore, root biomass (343 observations belonging to 118 clusters and 48 experiments) tended to be positively affected by BE addition (RR= 1.11, P=0.079, 95% C.I.: 0.99-1.24).

Subgroup or moderator analyses identified that crop type (maize, tomato or wheat) significantly affected BE effects (F=8.63; P<0.001). The effect of BE addition on yield was largest in tomato (RR = 1.27, P<0.001, 95% C.I.: 1.17-1.37), smaller in maize (RR=1.06, P<0.01, 95% C.I.: 1.02-1.11) and insignificant in wheat (RR=1.02, P=0.70, 95% C.I.: 0.93-1.11) (**Figure 3**). The same overall trend (tomato > maize > wheat) was observed in separately analyzed pot experiments, although the effect was less pronounced and not significant (P=0.067).

**Figure 4:**
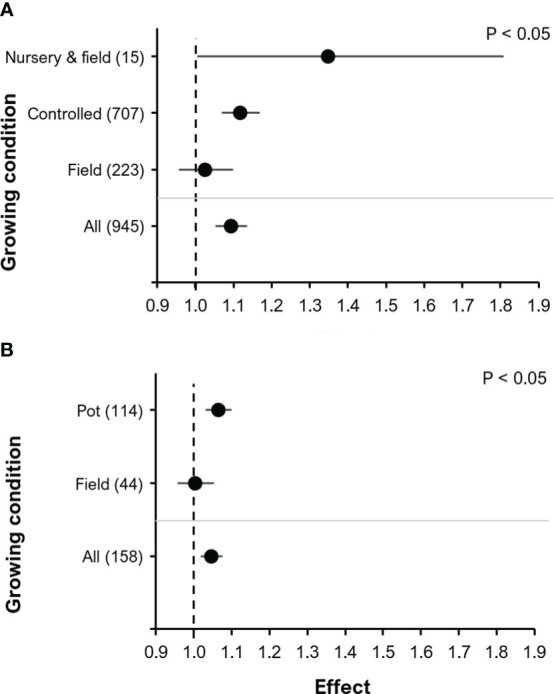
The effect of BE addition on yield according to the crop growing condition: Analysis including all observations **(A)**; Analysis only of maize in combination with the BE Proradix **(B)**. In **(A)** the combination “Nursery & field” is included which was only included in experiments with tomato (*Solanum lycopersicum L)*. For each part, the overall mean effect for the given subset of data is included at the bottom (All). For each growing condition, the number inside the brackets represent the number of observations included, the point indicates the mean effect while the horizontal error line represents the 95% C.I. The p-value indicates whether there was a significant effect of crop growing condition.

There was a significant effect of the growing condition on yield (F=3.13, P<0.05). The largest effect on yield (although highly variable) was observed in the controlled nursery (under greenhouse conditions) + field combination (RR=1.35, P<0.05, 95% C.I.: 1.00-1.81), it was smaller under controlled conditions (RR=1.12, P<0.001, 95% C.I.: 1.07-1.17) and only insignificant for field experiments (RR=1.03, P=0.48, 95% C.I.: 0.96-1.10) (**Figure 4A**). The controlled nursery + field combination was restricted to experiments with tomato. To further separate the effects on yield under controlled conditions versus field conditions, we performed a separate analysis using only results for the BE Proradix (*Pseudomonas* sp. DSMZ13134) tested in maize (**Figure 4B**), which was the crop/BE combination with the highest number of observations (n=158). A significant effect of the type of the growing condition (F=4.1, P<0.05) with a positive and significant effect of BE addition was seen under controlled conditions (RR=1.07, P<0.001, 95% C.I.: 1.03-1.10), but not under field conditions (RR=1.00, P=0.87, 95% C.I.: 0.96-1.05) (**Figure 4B**).

**Figure 5:**
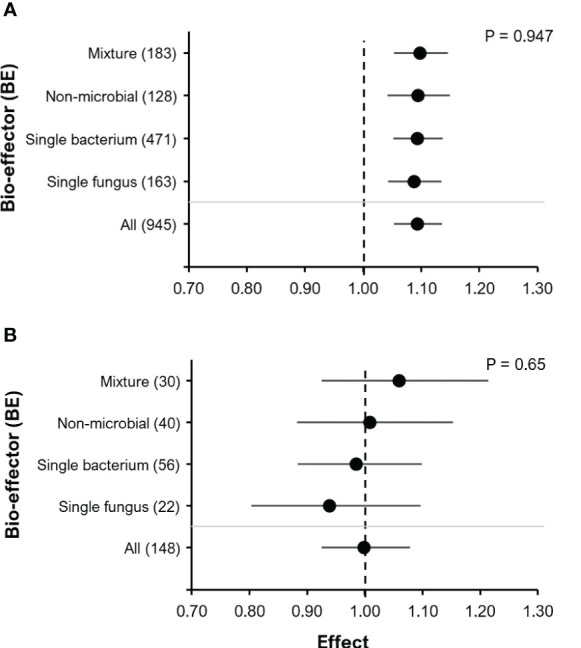
The effect of BE addition on yield according to BE type: Experiments without induced abiotic stress **(A)**; Experiments with induced abiotic stress (cold, drought/heat or salt stress) **(B)**. For each part, the overall mean effect for the given subset of data is included at the bottom (All). For each BE type, the number inside the brackets represent the number of observations included, the point indicates the mean effect while the horizontal error line represents the 95% C.I. The p-value indicates whether there was a significant effect of BE type.

Remarkably, all BE types (single bacteria, single fungi, non-microbials, mixtures) promoted very similar yield improvements without induced abiotic stress (P=0.947; **Figure 5A**), but with induced abiotic stress (salinity, drought and cold), which also increased experimental variability (P=0.65; **Figure 5B**).

**Figure 6:**
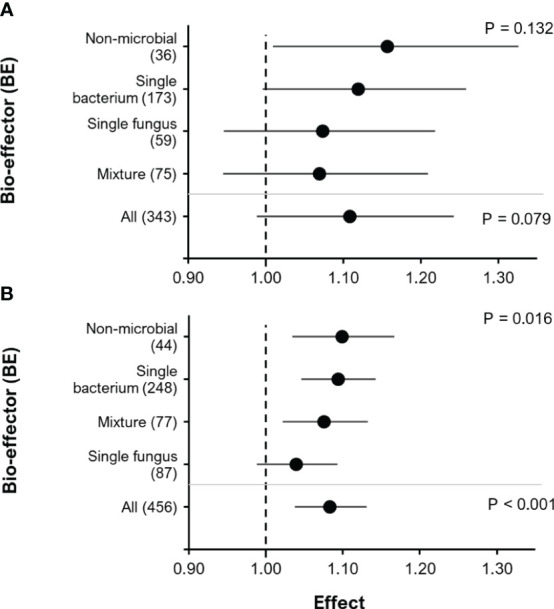
The effect of BE addition on root biomass **(A)** and P content in above-ground biomass **(B)** as a function of BE type. For each BE type, the number inside the brackets represent the number of observations included, the point indicates the mean effect while the horizontal error line represents the 95% C.I. The upper p-values indicate whether or not there was a significant effect of BE type on root biomass or P content in above-ground biomass. The lower p-values indicate whether or not there was an overall significant effect of BE addition for all BE types combined.

Unlike the positive effect of BE addition on yield, the effect on root biomass was not significant but showed only a positive trend (**Figure 6A**, RR= 1.11, P= 0.079; 95% C.I.: 0.99-1.24). Here, the effect on BE addition of root biomass tended to increase in the following order of BE type: Mixture< Single fungus< Single bacterium< Non-microbial. There was a significant positive effect of BE addition on P content in above-ground biomass (**Figure 6B**, RR= 1.083, P< 0.001; 95% C.I.: 1.037-1.13). The effect of BE type on above-ground biomass P content was also significant (F=3.5, P = 0.016). Comparably to the effect of BE type on root biomass, the effect of BE type on P content in above-ground biomass increased in the following order of BE type: Single fungus< Mixture< Single bacterium< Non-microbial.

**Figure 7:**
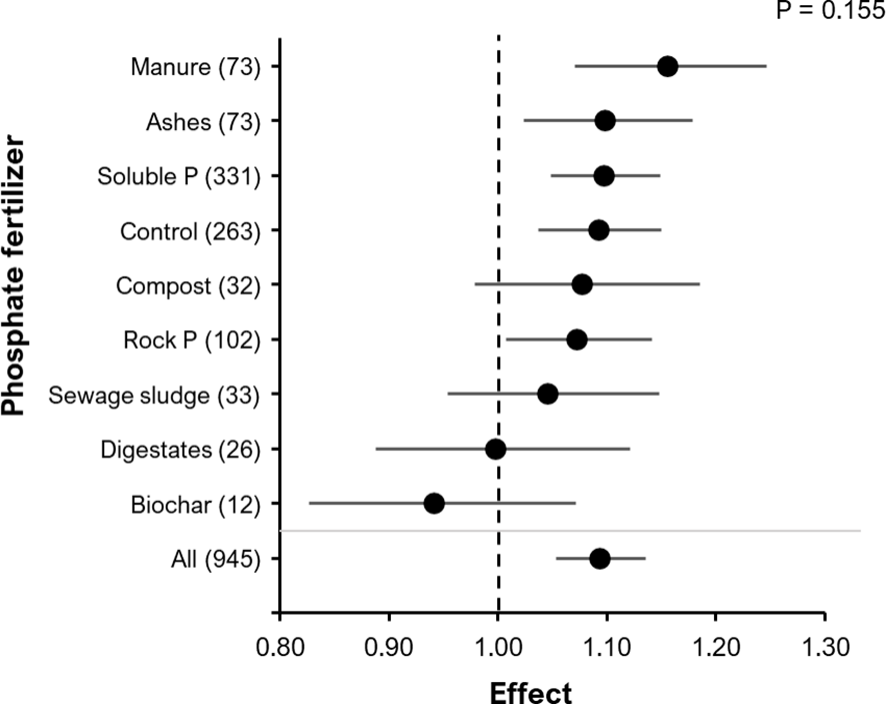
The effect of BE addition on yield as a function of the type of fertilizer added in the experiment Manure, guano hair-, feather-, meat and bone meal fertilizers were summarized in the category animal waste products (73); P = phosphate; Control = no P fertilizer applied). The analyzed data are on either: grain DM, fruit DM, fruit FM or shoot DM. A total of 945 observations from 290 clusters and 114 experiments were included in the analysis. For each phosphate fertilizer type, the number inside the brackets represent the number of observations included, the point indicates the mean effect while the horizontal line represents the 95% C.I. The p-value indicates whether there was a significant effect of phosphate fertilizer type.

Although the yield RR was not significantly affected by the type of fertilizer applied (manure, ashes, soluble P, control (no P fertilizer), municipal waste composts, rock P, sewage sludge, digestates, biochar) (P=0.155; **Figure 7**), animal waste products tended to have the strongest increase in the RR of BE addition, whereas Biochar even showed a negative trend. Comparing more specifically, different types of mineral and organic N-fertilization had no significant BE effect on the RR for yield, but a similar trend for highest performance of organic N fertilizers (**Supplementary Figure S2**). The application method for N-fertilizers (P = 0.96, **Supplementary Figure S3A**) or P-fertilizers (P= 0.39 **Supplementary Figure S3B**), including broadcast and localized placement techniques, did not have a significant effect on the RR of yield.

**Figure 8:**
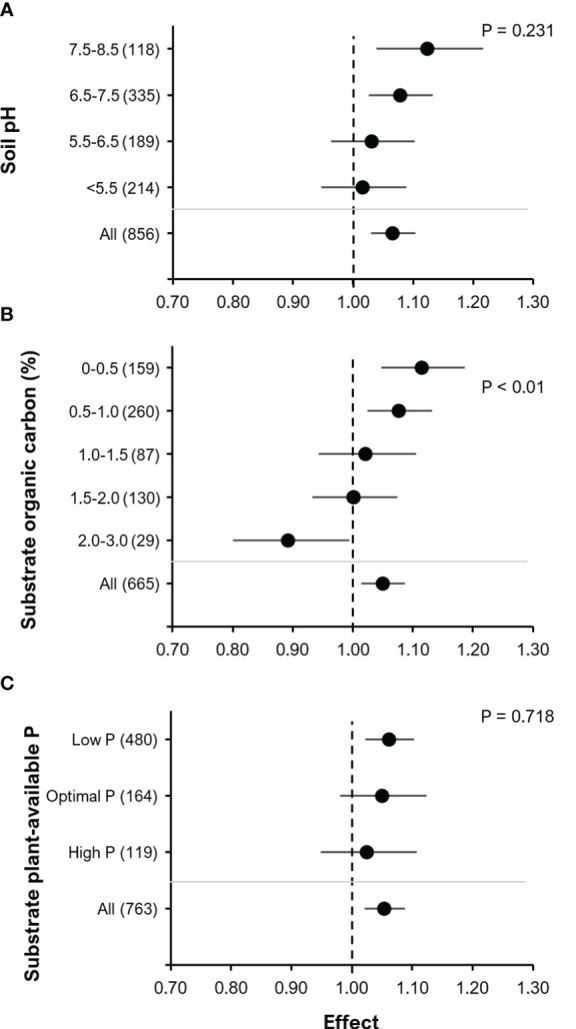
The effect of BE addition on yield as a function of the chemical properties of the soil or substrate used: pH **(A)**; % organic carbon concentration (% OC) * **(B)**; plant-available P** **(C)**. For each part, the overall mean effect for the given subset of data is included at the bottom (All). For each level of pH, % OC or available-P, the number inside the brackets represent the number of observations included, the point indicates the mean effect while the horizontal line represents the 95% C.I. The p-values indicate whether there was a significant effect of the chemical property. *The category OC 3.0 – 4.0% is has been excluded because it contains only three datasets (Effect= 1.079, 95% C.I. = 0.8062 – 1.445). ** Substrate plant-available P (mg P (kg dry soil)^-1^): Low P = P_CAL_< 45 or P_Olsen_< 20 or P_H20<_ 10; Optimal P = P_CAL_ 45 - 90or P_Olsen_ 20 - 40 or P_H20_ 10< 20; High P = P_CAL_ ≥ 91 or P_Olsen_ ≥ 40 or P_H20_ ≥ 20.

The effectiveness of BE addition on yield was related to substrate properties: pH, % organic carbon (%OC) and plant-available P (**Figure 8**). There was a tendency towards an increase in the effect of BE addition on yield with an increase in soil pH (F=1.44, P=0.23) i.e. BE addition tended to have the strongest effect in soils or substrates with an alkaline pH range of 7.5 – 8.5 (**Figure 8A**). We found a significant effect of %OC on the RR of BE addition to yield with an increase in BE effect on yield with decreasing substrate %OC (F= 3.74, P< 0.01; **Figure 8B**). Finally, there was also a trend of increasing RR of BE addition on yield with decreasing plant-available P in soils or substrates (**Figure 8C**) (F=0.33, P= 0.718).

## 4 Discussion

### 4.1 Main observations

In the experiments on improved nutrient acquisition, there was a positive relative effect of BE application on crop yield (9.3%) in comparison to the no BE control (**Figure 3**). The overall mean effect was not as strong as those of other meta-analyses (Herrmann et al., 2022; Schütz et al., 2018; Rubin et al., 2017), most likely because we considered all the results in our project, including also those lacking a positive growth response of BEs. This suggests the possibility of a considerable *publication bias* in more conventional meta-studies on the use of BEs. Rubin et al. (2017) reported a mean effect size of PGPRs of 28% on crop shoot biomass with the highest responsiveness under drought conditions. Similarly, Schütz et al. (2018) observed a mean effect size of approximately 16% on yield response of microbial inoculants applied in dry, tropical or continental climate zones, of which the smallest effect of 8.5% was recorded for trials in temperate continental climate zones. The overall effect size of 9.3% from this meta-analysis may be comparable to the latter (8.5%) possibly because the majority of the field observations in our study were also from temperate continental regions (**Figure 2**). This also points to a significant impact of the geo-climatic conditions, determining the efficiency of BE-assisted production strategies. In a global network meta-analysis, Herrmann et al. (2022) reported a 25% and 30% BE-induced increase in plant growth and yield, respectively. In comparison, only trials that were conducted within the EU and Israel were included in our current meta-analysis, whereas the study of Herrmann et al. was composed largely of trials conducted in lower- and upper middle-income countries such as India and China.

### 4.2 Pot versus field effects

Frequently, it is observed that effects of BEs are more reliably obtained under controlled laboratory conditions, where plants are grown in pots, compared to field experiments, where effects are more variable and often insignificant (Richardson and Simpson, 2011). The agronomic potential of biofertilizers for maize yield in pot experiments was higher than in field conditions (Schmidt and Gaudin, 2018). Similarly, we found that the mean effect size was higher in experiments conducted under controlled conditions compared to those conducted under field conditions (**Figure 4A**). The exception was the specific set of growing conditions starting with a controlled nursery and subsequent transplantation to the field, used for field-grown tomato, which had the largest mean BE effect on yield, but also the largest variability, potentially related to the lower number of observations. This large effect can most likely not be ascribed solely to the growing conditions. It might at least partially also be influenced by a crop-type induced effect of tomato, as described earlier. Nevertheless, a clear differentiation is not possible based on the available datasets. To further isolate the effect of pot experiments versus field experiments, we investigated the observations originating from the same crop and the same BE treatment. The BE/crop combination with the largest number of observations was the BE Proradix (*Pseudomonas* sp. DSMZ13134) applied in maize (**Figure 4B**). Similarly, to the complete dataset, we saw a significant effect in pot but not in field experiments. This is in accordance with the stronger yield increase observed for maize inoculated with *Pseudomonas* sp. under pot (24.9%) compared to field (13.8%) conditions (Schmidt and Gaudin, 2018). Particularly for rhizosphere-microbial BEs investigated in our study, efficient root colonization is a prerequisite for the expression of beneficial BE effects (Dobbelaere et al., 2001; Berg et al., 2021). This is achieved more easily under controlled conditions, excluding external stress factors with negative impact on vitality of inoculants, root growth and activity, which did not apply for field conditions. Moreover, pot experiments allowed repeated inoculations of small, densely rooted-soil volumes during the culture period, known to promote root colonization (Nkebiwe et al., 2017). This is not the case for most field experiments, where seed treatments or seeding row inoculations at the begin of the culture period are frequently the only technically and economically feasible options. However, the potential benefits on seedling establishment and early growth do not necessarily translate into comparable yield effects under field conditions (Mpanga et al., 2019b; Dobbelaere et al., 2001). In contrast to our finding that the effect of BEs on crop yield under field conditions was not significant, in another meta-analysis, Li et al. (2022) showed an overall yield increase of 17.9% attributed to biostimulants applied to open field crops. This result is very promising and the difference to the results of this paper can be explained by the type of biostimulants applied. Whereas only 128 of the 945 datasets used in our study is from the application on non-microbial biostimulants (13.5%), the meta-analysis by Li et al. (2022) was focused solely on non-microbial biostimulants (100%). This again highlights efficient root colonization as a prerequisite for the expression of beneficial BE effects for microbial biostimulants under field conditions (Dobbelaere et al., 2001; Berg et al., 2021).

### 4.3 Crop-specific effects

We observed a larger mean BE effect in tomato compared to the two monocot crops (**Figure 3**). Rubin et al. (2017) also reported a difference in the effects of PGPR in different crops. They found strong effects (~40% increase) in forbs, legumes and C4 grasses on shoot biomass and insignificant effects in C3 grasses. This is to some extent supported by our data, as we did not observe an overall positive effect of BE addition on the yield of wheat (a C3 grass), whereas we found a significant positive effect in the yield of maize (a C4 grass). The lack of effect in wheat in the present analysis is in accordance with a series of field trials reported by Karamanos et al. (2010), in which inoculation with *Penicilium bilaii* resulted in an increase in wheat P uptake in only a few cases (4 out of 33 experiments). As in our analysis, Schütz et al. (2018) observed stronger effects of BE addition in vegetables as compared to cereals. Similarly, Rho et al. (2018) observed C4 plants to be more responsive to endophyte inoculations than C3 plants when subjected to drought stress conditions. This has also been reported for diazotrophic bacteria used as inoculants (Dobbelaere et al., 2001). This may be attributed to the higher efficiency of C4 photosynthesis under tropical and subtropical conditions, mediating a more efficient carbon supply to microbial inoculants (Bennett et al., 2020). In an unweighted meta-analysis of published studies, also Megali et al. (2015) found large plant-specific differences in the effect of Effective Microorganisms^®^. Only considering humic substances as BEs, Rose et al. (2014) observed a higher responsiveness of monocots compared to dicots in terms of shoot dry weight increase, while the opposite was true for root dry weight. Focusing on the effects of AMF, also strong growth promotion effects in wheat can be observed (Omar, 1997; Kucey, 1987). Apart from differences between species, there may also be important differences between the effect in different cultivars as shown by Harman et al (Harman, 2006). for *Trichoderma* in maize or by Valente et al. (2020) for *Pseudomonas* in wheat.

### 4.4 BE-specific effects

Interestingly, there were no significant differences between BE categories, all with a very similar effect size (9-10%) (**Figure 5**). The meta-analysis by Herrmann et al. (2022) also found no significant differences between BE categories. Overlapping beneficial effects reported for many microbial and non-microbial BEs, based on root growth promotion, scavenging of reactive oxygen species (ROS) or effects on hormonal balances (van Oosten et al., 2017; Sani and Yong, 2022), might partially explain the observed similarities. Furthermore, testing only the most promising BE-crop combinations in the field, based on the results from greenhouse experiments, represents a possible experimental bias, which could explain why in our case the RR differed not as strong as it might have been expected when compared to other studies (Schmidt and Gaudin, 2018; Ansari et al., 2015; Stamford et al., 2007).

In addition to the yield benefits, the application of BE led to a non-significant trend for increased root biomass production (**Figure 6A**), which was associated to improved BE-induced nutrient acquisition was reflected by increased P accumulation in the shoot tissues (**Figure 6B**), This may reflect a contribution of BE-mediated root growth promotion to P acquisition as demonstrated in numerous studies on BE functions, conducted within the project (Bradáčová et al., 2019b; Mpanga et al., 2019b; Mpanga et al., 2018; Mpanga et al., 2019a; Weber et al., 2018; Eltlbany et al., 2019), although root length rather than root biomass would be a more reliable indicator in this context. By contrast, mobilization of sparingly soluble P sources by microbial inoculants could not be identified as an important mechanism contributing to P acquisition in most experiments addressed in this meta-analysis (Bradáčová et al., 2019b; Mpanga et al., 2019b; Mpanga et al., 2019a; Lekfeldt et al., 2016; Thonar et al., 2017). This was confirmed also on a more general basis in a recent review covering the scientific literature on P solubilizing microorganisms as plant inoculants since 1948, coming to a final conclusion that despite significant long-term contributions of native Phosphate Solubilizing Microorganism (PSM) populations in soil to P cycling in ecosystems, PSM inoculants do not mobilize sufficient P to change the crops’ nutritional environment under field conditions (Raymond et al., 2021).

When comparing BE responses with other meta-studies, we found that single bacterial strains (**Figure 5A**) showed a lower mean effect size (9.3%) in our study as compared to that of Rubin et al. (2017), who reported an effect size of 32%. However, the single bacteria effect size of 9.3% is comparable to the results obtained by Veresoglou & Menexes (Veresoglou and Menexes, 2010), who observed increases in wheat grain yield of 8.9% after inoculation with *Azospirillum* sp. The effect size for shoot yield with the same inoculum was higher 17.8%. In comparison to our observations for single fungal strains (8.8%), Leggett et al. (2015) found a more moderate effect of up to 3.7% for the inoculation with *Penicilium bilaii* on the yield in maize (**Figure 5A**).

Some authors have observed a larger effect when more than one microbial isolate or combinations of microbial and non-microbial BEs were applied (BE consortia) (Bradáčová et al., 2019b; Kumar et al., 2016; Singh et al., 2014). Rubin et al. (2017) observed a superior performance of microbial consortia in enhancing shoot dry weights across several crop species. Also, the meta-study of Schütz et al. (2018) found that a PGPM consortia composed of N-fixers and P-solubilizers were more effective than single inoculations with P-solubilizers.

In contrast, we did not observe a larger effect of using BE combinations as opposed to single BE products in the experiments on improved nutrient acquisition (**Figure 5A**). However, under conditions with induced abiotic stress (cold, drought/heat or salt stress), a trend of increasing RR according to the BE type was recorded in the order single fungus< single bacterium< non-microbial< mixture (**Figure 5B**). The low and partially even negative mean effect size of single strain microbial inoculants in this case may reflect the well-documented sensitivity of many beneficial plant-microbial interactions to stress conditions acting during the establishment phase (Backer et al., 2018); which may be compensated by BE combinations with complementary or synergistic stress-protective functions (Bradáčová et al., 2019b; Moradtalab et al., 2020).

### 4.5 Effect of fertilizers

Special emphasis was put on P acquisition and fertilizer-based products of organic and inorganic waste recycling. Although there was no significant effect of P (**Figure 7**) or N fertilizers (**Supplementary Figure S1**) on the BE effect size on yield, we observed trends among different fertilizer types. Largest BEs effects were obtained in combination with fertilizers derived from N and P rich animal waste products, such as manure-based fertilizers, hair-, feather-, meat- and bone-meals. This was confirmed particularly for microbial BEs in numerous studies conducted within the project (Mpanga et al., 2018; Thonar et al., 2017; Li et al., 2018; Vinci et al., 2018b; Vinci et al., 2018a; Bradáčová et al., 2019a; Cozzolino et al., 2021). The supply of organic C from fertilizers might have promoted this effect since many PGPMs are characterized as fast-growing copiotrophic microorganisms with a high demand for easily available carbon sources. Accordingly, Windisch et al. (2021) demonstrated that low rhizosphere abundance of PGPMs in lettuce was associated with limited availability of low molecular weight sugars in the rhizosphere soil solution. Moreover, due to high N and P availability, the respective organic fertilizers could provide a starter fertilization effect, which is a well-documented measure to promote the establishment of symbiotic plant-microbial interactions (Bittman et al., 2006; Chekanai et al., 2018), and likely applies similarly to other PGPMs. Root growth promotion, interactions with the plant hormonal status and mineralization of nutrients in the organic fertilizers induced by the microbial inoculants and/or related soil microbiome shifts are potential modes of action in this context (Richardson, 2001; Eltlbany et al., 2019; Cozzolino et al., 2021). However, we did not find evidence to suggest a larger RR with the application of organic fertilizers in general, which again indicates that the interaction of many factors influences the effectiveness of the BEs.

N-fertilizer form has been shown to influence the effects of BEs on crop yield with stabilized ammonium, leading to the highest increases in yield related with improved P acquisition (Bradáčová et al., 2019b; Mpanga et al., 2019b; Nkebiwe et al., 2017; Mpanga et al., 2018; Mpanga et al., 2019a; Nkebiwe et al., 2016b; Mpanga et al., 2020). The effect of ammonium on BE-induced yield increase could not be captured adequately by this meta-analysis (**Supplementary Figure S1**) probably because few observations and large variability was associated with the category stabilized ammonium fertilizer) in comparison to other mineral N-fertilizer forms (n = 800). Moreover, the ammonium effect was limited to soils with low P availability and moderate pH buffering capacities, which would not counteract ammonium-induced rhizosphere acidification by plant roots (Bradáčová et al., 2019b; Mpanga et al., 2020).

Although there is some evidence that localized placement of root growth-stimulating stabilized ammonium fertilizers in soil may enhance root colonization of microbial BEs and improve yield (Nkebiwe et al., 2017; Bradáčová et al., 2019a), N-fertilizer (**Supplementary Figure S2A**) or the P fertilizer (**Supplementary Figure S2B**) application method did not influence the effect of BE addition. There was large variability in the effect sizes of the different fertilizer application methods. Regarding alternative P fertilizer sources, there is some evidence that the combination of a sparingly soluble P fertilizer like rock phosphate and compost increases P bio-availability (Redel et al., 2019), which may be further improved with BE addition. Additionally, direct use of sewage sludge showed a low RR on yield after BE addition (**Figure 7**). Alternatively, pyrolyzed sewage sludge (ash) may be used to partially replace rock phosphate in in the production of P fertilizers to improve its plant availability (You et al., 2021). This would also contribute to closing the P cycle and alleviating environmental problems associated with high P losses through unrecycled waste materials.

### 4.6 Effect of soil properties

A trend towards an increased yield response to BE application with increasing soil pH suggest that P availability, as influenced by soil pH, may play an important role in the mode of action (Egamberdiyeva, 2007). The majority of observations in our study comprised soils with neutral to slightly alkaline pH, limiting P solubility by precipitation of Ca-phosphates and this applied also for many of the tested P fertilizers, such as superphosphate, rock-phosphates, ashes and slags. This may represent a major nutrient limitation mitigated by BE applications (**Figure 8A**). In addition, Rousk et al. (2009) observed an inhibiting effect of decreasing soil pH on bacterial activity. This may explain why we observed an increase in crop yield with effect of BE addition only at elevated soil pH (6.5 – 8.5) but not at low pH (<6.5) conditions. Also, Schütz et al. (2018) found a positive relationship between soil pH and yield response in their meta-analysis for P solubilizers in combination with N fixers, while for N fixers alone and P solubilizers alone, no and only a weak trend was found respectively. For AMF, there was a tendency towards a bell-shaped curve and related this to an increased availability of macronutrients at an intermediate pH (~7.5). Investigating the effect of the fungus *P. bilaii* in maize, Leggett et al. (2015) did not find a significant correlation between yield response and soil pH. In contrast, Sánchez-Esteva et al. (2016) reported that the effect of *P. bilaii* on wheat plant was affected by soil pH. So, it seems that soil pH might affect the size of BE effects but that the impact of pH seems to depend on other factors such as crop type and their inherent nutrient acquisition strategies.

In accordance with the results obtained in the meta-analysis of Schütz et al. (2018), we observed a decrease in the RR for yield with an increase in the soil/substrate organic carbon content (%OC, **Figure 8B**). This might be related to a general increase in microbial abundance, diversity and activity with increasing soil organic carbon/matter status as revealed by the meta-analysis of Lori et al. (2017). An increase in soil organic carbon status is reported to increase populations of plant beneficial microorganisms (Francioli et al., 2016). This might in turn hamper the establishment of introduced microorganisms due to increased competition from the native microbial community (Paul, 2016). Moreover, the expected benefits of BE applications may be triggered already by the higher abundance of indigenous beneficial microbes in soils with high organic carbon content. The stimulatory effects of organic carbon supply on microbial BEs might at least partially explain also the observed benefits of combined application with selected organic fertilizers based on manures and animal waste products (**Figure 7**).

In contrast to Schütz et al. (2018), who observed significant effects of the level of plant-available soil P on the BE RR of yield, we could not find a significant effect of this soil property. Schütz et al. (2018) found that plant-available soil P status (extraction and analytical methods: Olsen, Bray, Mehlich, and AB DTPA- ammonium bicarbonate-diethylenetriaminepentaacetic acid) triggering best performance differ depending on the type of biofertilizers applied. N fixers preferred higher soil P concentrations than P solubilizers. In all cases, BE responses declined at the lowest P availability, but also at higher P levels. Since we evaluated the effect of plant available soil P across different types of BEs on non-legumes, we might have masked or missed BE type specific effects for legumes as reported by Schütz et al. (2018). Nonetheless, similar to Schütz et al. (2018), we still observed a trend of decreasing RR with increasing soil plant-available P status (**Figure 8C**). This is well explained if under elevated plant available P levels plants can independently acquire sufficient amounts of P for optimal growth and are then less dependent on the support by BEs for nutrient acquisition. However, our data on BE relationships with native soil available P are not directly comparable, since Schütz et al. (2018) considered both, native soil available P and fertilizer P. The trend we observed of an increasing effect of BE addition on yield with decreasing plant-available soil P may be linked to BE-assisted mobilization of naturally inherent soil P or legacy soil P, which may constitute a substantial amount of P after a history of P-fertilizer application in agricultural soils (Yu et al., 2021).

Given the elaborate and transdisciplinary nature of the project (**Figure 2**), a comprehensive list of 107 biostimulants (microbial and nonmicrobial, **Supplementary Table S2**) were evaluated on 24 crop*cultivar combinations (**Supplementary Table S3**), in 94 soils (**Table S5**) and fertilized with 145 fertilizers (**Supplementary Table S4**) in 136 different experiments (**Supplementary Table S1**), explicitly excluding a publication bias. This comprehensive design was well-suited to generate overall summary effects as a first overview to determine the global effectiveness of biostimulants under different conditions. However, a potential limitation of this approach was the high degree of heterogeneity brought in, which made it sometimes challenging to uncover statistically significant differences between levels of different moderators/groupings. To reduce the variability, it may be recommended for future studies to focus now on fewer, more defined classes of biostimulants evaluated with more repetitions and a more specific focus on selected physico-chemical soil or geoclimatic conditions. This may also be well suited for more targeted mode of action studies in addition to agronomic evaluation with the final goal to define conditions and indicators for successful application of biostimulants in agricultural practice.

## 5 Conclusion and outlook

Bio-effector-based production strategies can offer perspectives to improve plant productivity without acting as direct nutrient sources (Halpern et al., 2015; Du Jardin, 2015). Nevertheless, our study identified limitations for their successful agronomic use. The global biofertilizers market size steadily increased in recent years with several new commercial products emerging every year. In 2019, it was valued at USD 1.0 billion and is anticipated to witness a compound annual growth rate of 12.8% from 2020 to 2027 (Grant review research, 2020). Our results suggest that all BEs stimulate plant growth to a similar extent under conditions representative for European agriculture. Horticultural crops, such as tomato, grown under greenhouse conditions at least during a nursery phase used for BE inoculation are most promising. To a limited extent, similar benefits were recorded for maize as a field crop, especially when the soil is characterized by a low organic carbon content and a neutral to alkaline pH value. BEs appeared to exert strongest effects when combined with manures and organic N fertilizers and their efficiency declined with increasing soil nutrient status. The similar overall performance of microbial and non-microbial BEs (i.e. seaweed/plant extracts and humic acids) offers flexibility for application strategies and points to improved root growth as a common stimulation mechanism for crop growth. Rhizosphere microbial BEs seem most promising as starter applications promoting seedling establishment and early growth, while non-microbial BEs can be applied more flexible by soil drenching and also as foliar sprays in later stages of the culture period. As all BEs had a similar growth effect, this potentially indicates that common physiological plant growth stimulation mechanisms were involved. Combinations of different BEs with complementary properties may provide an additional option for improved performance under conditions of mild cold stress, drought or salinity, but stronger stress appears to impair beneficial effect.

## Data availability statement

The raw data supporting the conclusions of this article will be made available by the authors through the permanent repository PANGAEA (https://www.pangaea.de/) without undue reservation.

## Author contributions

PN: Conceptualization, Data curation, Formal analysis, Investigation, Methodology, Software, Supervision, Validation, Visualization, Writing – original draft, Writing – review & editing. JL: Conceptualization, Data curation, Formal analysis, Investigation, Methodology, Software, Supervision, Validation, Visualization, Writing – original draft, Writing – review & editing. SS: Conceptualization, Investigation, Supervision, Writing – original draft, Writing – review & editing. CT: Investigation, Supervision, Writing – review & editing. PM: Supervision, Writing – review & editing. AB-T: Supervision, Writing – review & editing. MH: Investigation, Writing – review & editing. BB: Supervision, Writing – review & editing. KB: Investigation, Writing – review & editing. PC: Investigation, Writing – review & editing. KC: Investigation, Writing – review & editing. VC: Supervision, Writing – review & editing. ED: Investigation, Writing – review & editing. SD: Investigation, Writing – review & editing. JG: Supervision, Writing – review & editing. AL: Investigation, Writing – review & editing. BG-M: Investigation, Writing – review & editing. EK: Supervision, Writing – review & editing. FK: Investigation, Writing – review & editing. ZK: Investigation, Writing – review & editing. MK: Supervision, Writing – review & editing. FM: Investigation, Writing – review & editing. GT: Investigation, Writing – review & editing. NM: Investigation, Writing – review & editing. AP: Supervision, Writing – review & editing. AM: Supervision, Writing – review & editing. DN: Investigation, Writing – review & editing. MS: Supervision, Writing – review & editing. KJ: Investigation, Writing – review & editing. CF: Supervision, Writing – review & editing. AF: Investigation, Writing – review & editing. GP: Supervision, Writing – review & editing. KL: Supervision, Writing – review & editing. BT: Investigation, Writing – review & editing. PT: Supervision, Writing – review & editing. IM: Investigation, Writing – review & editing. NW: Investigation, Writing – review & editing. MW: Funding acquisition, Investigation, Supervision, Writing – review & editing. UY: Supervision, Writing – review & editing. JM: Supervision, Writing – review & editing. TM: Funding acquisition, Supervision, Writing – review & editing. GN: Funding acquisition, Supervision, Writing – original draft, Writing – review & editing. UL: Funding acquisition, Supervision, Writing – original draft, Writing – review & editing. AdN: Conceptualization, Validation, Writing – original draft, Writing – review & editing.

## Supplementary material

Supplementary Table 1 Overview table with experiments and clusters (control groups) within experiments

Supplementary Table 2 List of BE treatments applied. Both single treatments and combinations. Includes information on contents and producers.

Supplementary Table 3 List of crops included + cultivars + information on suppliers

Supplementary Table 4 List of P and N fertilizers applied

Supplementary Table 5 List of soils used with basic info on pH, P content, texture, %C.
