# Supplementary material for: Effectiveness of bio-effectors on maize, wheat and tomato performance and phosphorus acquisition from greenhouse to field scales in Europe and Israel: a meta-analysis
Source: Front Plant Sci. 2024 Apr 2;15:1333249. doi: 10.3389/fpls.2024.1333249 (PMC11020074; doi:10.3389/fpls.2024.1333249)
Supplement: Supplementary Table 1 — Overview table with experiments and clusters (control groups) within experiments [file DataSheet_1.pdf]

Figures for supplementary materials

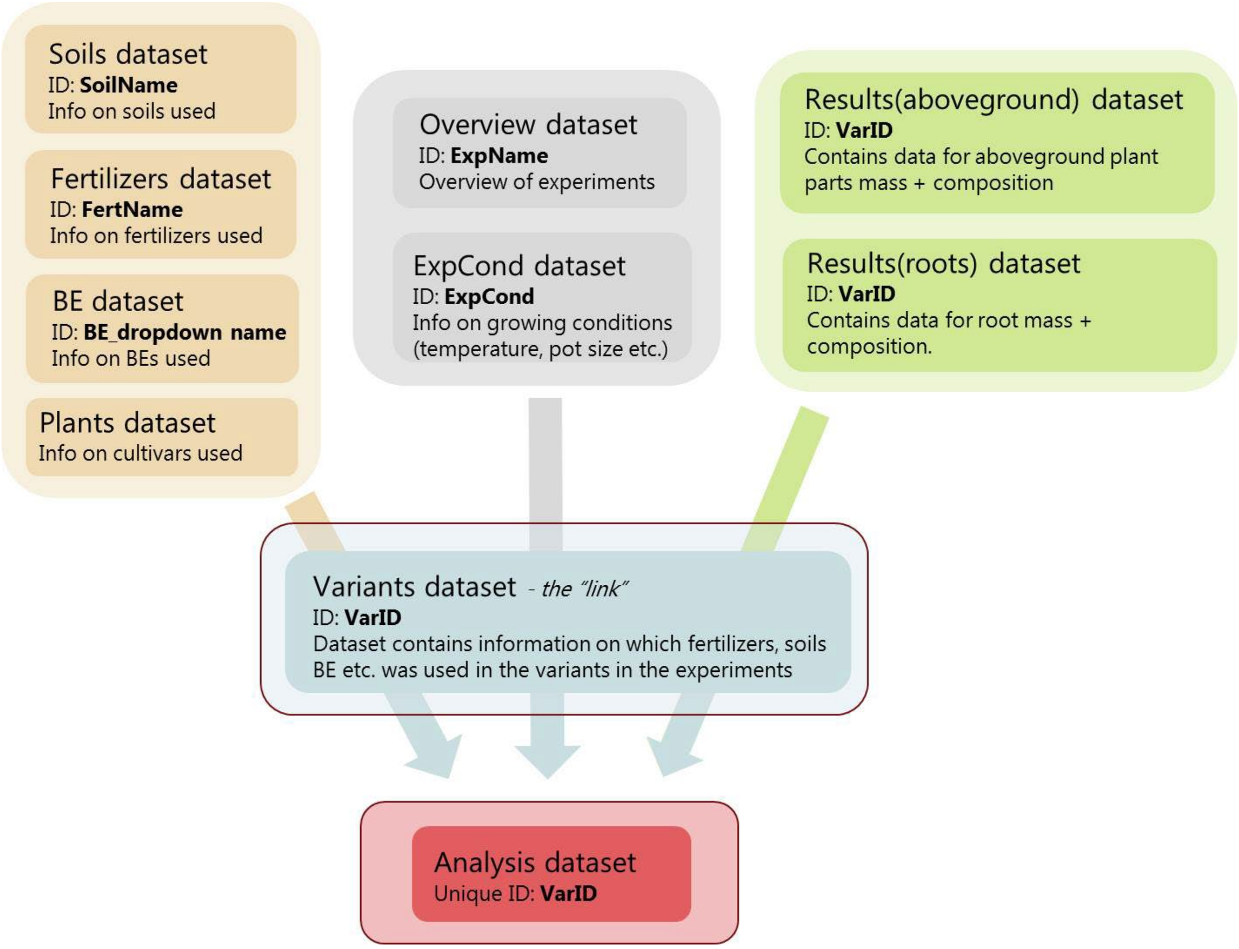

Figure S1: Overview of the structure of the BIOFECTOR database.

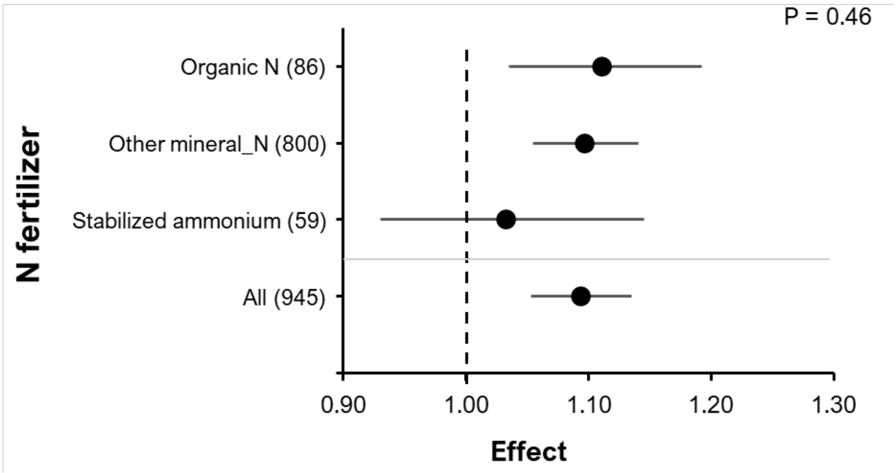

Figure S2. The effect of BE addition on yield (either grain DM, fruit DM, fruit FW or shoot DM) within all experiments as a function of N fertilizer type. A total of 945 observations from 290 clusters were included in the analysis. For each N-fertilizer type, the number inside the brackets represents the number of observations included, the point indicates the mean effect while the horizontal line represents the 95% C.I. The p-value indicates whether there was a significant effect of N-fertilizer type.

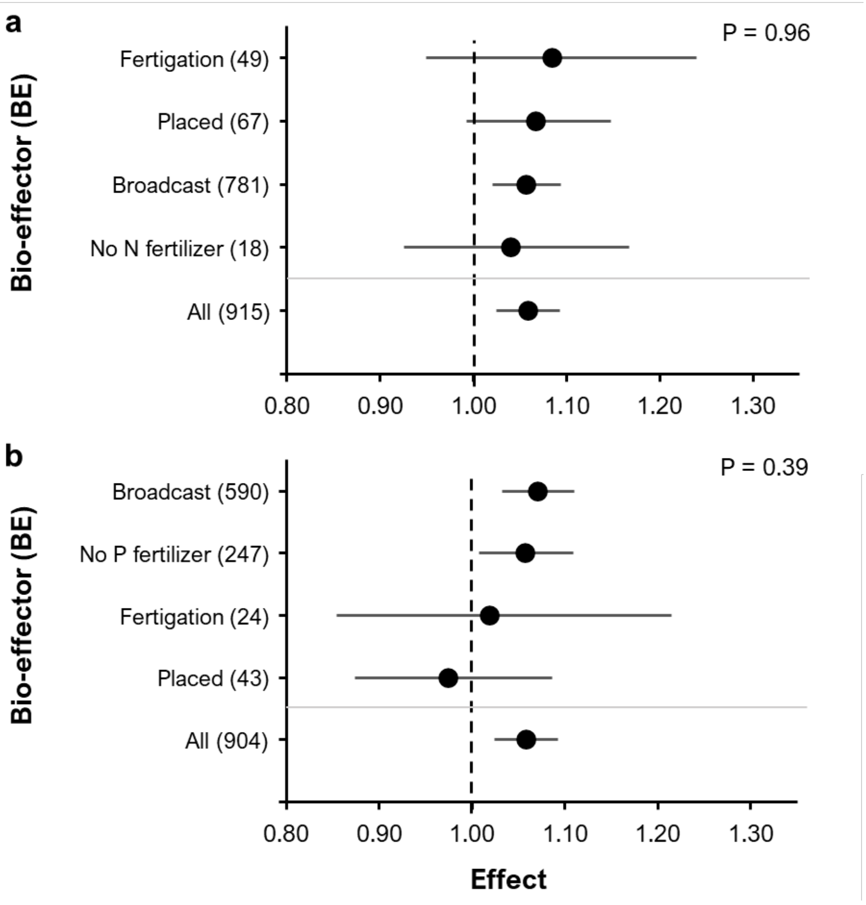

Figure S3. The effect of BE addition on yield (either grain DM, fruit DM, fruit FW or shoot DM) as a function of fertilizer application method: Application method for N-fertilizers (**a**) and P-fertilizers (**b**).For each fertilizer application method, the number inside the brackets represents the number of observations included, the point indicates the mean effect while the horizontal line represents the 95% C.I. The p-value indicates whether or not there was a significant effect of fertilizer application method.
