# Supplementary material for: Effectiveness of bio-effectors on maize, wheat and tomato performance and phosphorus acquisition from greenhouse to field scales in Europe and Israel: a meta-analysis
Source: Front Plant Sci. 2024 Apr 2;15:1333249. doi: 10.3389/fpls.2024.1333249 (PMC11020074; doi:10.3389/fpls.2024.1333249)
Supplement: Supplementary Table 2 — List of BE treatments applied. Both single treatments and combinations. Includes information on contents and producers. [file DataSheet_2.pdf]

Table S1: List of Experiments

Experiment	Cluster	Type	Crop	Soil	Nitrogen		Phosphorous		no. of BE
**Experiment**	**Cluster**	**Type**	**Crop**	**Soil**	**N source**	**N form**	**P source**	**P form**	**no. of BE**
ABI_2015	ABI_2015_A	Field	Maize	Sanitz_B	HAST-NP_A	Synthetic N	NP_A	Soluble P	5
ABI_2016	ABI_2016_A	Field	Maize	Sanitz_B	Sulfammo32-ActivN	Synthetic N	ActivN	Soluble P	5
AFBI_2014a	AFBI_2014a_A	Field	Barley	Newforge	CAN_C	Synthetic N	noP	Control	4
AFBI_2014b	AFBI_2014b_A	Field	Barley	Poyntzpass	CAN_C	Synthetic N	Phosphate	Soluble P	4
AFBI_2015c	AFBI_2015c_A	Field	Wheat	Newforge	CAN_C	Synthetic N	noP	Control	4
AFBI_2015d	AFBI_2015d_A	Field	Wheat	Poyntzpass	CAN_C	Synthetic N	Phosphate	Soluble P	4
AFBI_2016a	AFBI_2016a_A	Field	Wheat	Newforge	CAN_C	Synthetic N	noP	Control	4
AFBI_2016b	AFBI_2016b_A	Field	Wheat	Poyntzpass	CAN_C	Synthetic N	Phosphate	Soluble P	4
ARO_2013	ARO_2013_A	Pot	Tomato	Ramat	Solution_I	Synthetic N	SP_A	Soluble P	1
ARO_2013	ARO_2013_B	Pot	Tomato	Ramat	Solution_I	Synthetic N	SP_A	Soluble P	1
ARO_2013	ARO_2013_C	Pot	Tomato	Ramat	CompMan_I	Organic N	CompMan_I	Manure	1
ARO_2013	ARO_2013_D	Pot	Tomato	Ramat	CompMan_I	Organic N	CompMan_I	Manure	1
ARO_2014	ARO_2014_A	Pot	Tomato	Ramat	Solution_I	Synthetic N	TSP_E	Soluble P	4
ARO_2014	ARO_2014_B	Pot	Tomato	Ramat	Solution_I	Synthetic N	TSP_E	Soluble P	4
ARO_2014	ARO_2014_C	Pot	Tomato	Ramat	Solution_I	Synthetic N	TSP_E	Soluble P	4
ARO_2014	ARO_2014_D	Pot	Tomato	Ramat	Solution_I	Synthetic N	TSP_E	Soluble P	4
ARO_2015	ARO_2015_A	Pot	Tomato	Ramat	Solution_I	Synthetic N	TSP_E	Soluble P	3
ARO_2015	ARO_2015_B	Pot	Tomato	Ramat	Solution_I	Synthetic N	TSP_E	Soluble P	3
ARO_2015	ARO_2015_C	Pot	Tomato	Ramat	Solution_I	Synthetic N	TSP_E	Soluble P	3
ARO_2017	ARO_2017_A	Field	Tomato	Ramat	Solution_I	Synthetic N	TSP_E	Soluble P	4

Experiment	Cluster	Type	Crop	Soil	Nitrogen		Phosphorous		no. of BE
Experiment	Cluster	Type	Crop	Soil	N source	N form	P source	P form	no. of BE
ARO_2017	ARO_2017_B	Field	Tomato	Ramat	Solution_I	Synthetic N	TSP_E	Soluble P	4
ARO_2017	ARO_2017_C	Field	Tomato	Ramat	Solution_I	Synthetic N	TSP_E	Soluble P	4
AUAS_2013	AUAS_2013_A	Pot	Tomato	Chernozem	CAN_A	Synthetic N	noP	Control	18
AUAS_2015	AUAS_2015_A	Pot	Tomato	Chernozem	CAN_A	Synthetic N	noP	Control	18
AUAS_2016	AUAS_2016_A	Pot	Tomato	Chernozem	NovaTec	Ammonium	noP	Control	9
AUAS_2016	AUAS_2016_B	Pot	Tomato	Chernozem	CAN_A	Synthetic N	noP	Control	9
BUAS_2013a	BUAS_2013a_A	Pot	Tomato	Romania_mix_A	OrgFert_B	Organic N	OrgFert_B	Organic fertilizer	3
BUAS_2013b	BUAS_2013b_A	Field	Tomato	Romania_A	Multi-K	Synthetic N	noP	Control	3
BUAS_2013c	BUAS_2013c_A	Field	Maize	Romania_B	NPK_A	Synthetic N	NPK_A	Soluble P	7
BUAS_2013d	BUAS_2013d_A	Field	Maize	Romania_C	NPK_A	Synthetic N	NPK_A	Soluble P	7
BUAS_2014a	BUAS_2014a_A	Pot	Tomato	Romania_mix_B	OrgFert_B	Organic N	OrgFert_B	Organic fertilizer	3
BUAS_2014b	BUAS_2014b_A	Field	Tomato	Romania_D	Multi-K	Synthetic N	noP	Control	3
BUAS_2014c	BUAS_2014c_A	Field	Wheat	Romania_E	Man_E-NPK_A	Synthetic N	Man_E-NPK_A	Soluble P	5
BUAS_2014d	BUAS_2014d_A	Field	Wheat	Romania_F	Man_E-NPK_A	Synthetic N	Man_E-NPK_A	Soluble P	5
BUAS_2015a	BUAS_2015a_A	Pot	Tomato	Romania_mix_C	OrgFert_C	Organic N	OrgFert_C	Organic fertilizer	3
BUAS_2015b	BUAS_2015b_A	Field	Tomato	Romania_G	Multi-K	Synthetic N	noP	Control	5
BUAS_2015c	BUAS_2015c_A	Field	Wheat	Romania_H	Man_F-NPK_A	Synthetic N	Man_F-NPK_A	Soluble P	6
BUAS_2015d	BUAS_2015d_A	Field	Maize	Romania_I	Man_F-NPK_A	Synthetic N	Man_F-NPK_A	Soluble P	6
BUAS_2016a	BUAS_2016a_A	Pot	Tomato	Romania_mix_D	OrgFert_D	Organic N	OrgFert_D	Organic fertilizer	5
BUAS_2016b	BUAS_2016b_A	Field	Tomato	Romania_J	Multi-K	Synthetic N	noP	Control	5
BUAS_2016c	BUAS_2016c_A	Pot	Tomato	Romania_mix_D	OrgFert_D	Organic N	noP	Control	5

Experiment	Cluster	Type	Crop	Soil	Nitrogen		Phosphorous		no. of BE
Experiment	Cluster	Type	Crop	Soil	N source	N form	P source	P form	no. of BE
BUAS_2016c	BUAS_2016c_B	Pot	Tomato	Romania_mix_E	DuraTec	Ammonium	DuraTec	Soluble P	5
BUAS_2016d	BUAS_2016d_A	Field	Wheat	Romania_K	Man_G-NPK_A	Synthetic N	Man_G-NPK_A	Soluble P	6
BUAS_2016e	BUAS_2016e_A	Field	Maize	Romania_L	Man_G-NPK_A	Synthetic N	Man_G-NPK_A	Soluble P	6
CUB_2014a	CUB_2014a_A	Pot	Tomato	CUB_A	CAN_A	Synthetic N	TSP_A	Soluble P	3
CUB_2014b	CUB_2014b_A	Field	Tomato	CUB_A	CAN_A-OrgFert_A	Synthetic N	OrgFert_A	Manure	6
CUB_2015a	CUB_2015a_A	Pot	Tomato	CUB_B	CAN_A	Synthetic N	TSP_A	Soluble P	5
CUB_2015b	CUB_2015b_A	Field	Tomato	CUB_B	OrgFert_A	Organic N	OrgFert_A	Manure	6
CUB_2016a	CUB_2016a_A	Field	Tomato	CUB_C	OrgFert_A	Organic N	OrgFert_A	Manure	3
CULS_2013a	CULS_2013a_A	Pot	Maize	Humpolec_A	CalciumNitrate_A	Synthetic N	noP	Control	2
CULS_2013a	CULS_2013a_B	Pot	Maize	Humpolec_A	CalciumNitrate_A	Synthetic N	TSP_B	Soluble P	2
CULS_2014a	CULS_2014a_A	Pot	Wheat	Humpolec_B	AN_A-BioAsh_B	Synthetic N	BioAsh_B	Ashes	2
CULS_2014a	CULS_2014a_B	Pot	Wheat	Humpolec_B	AN_A-BioAsh_A	Synthetic N	BioAsh_A	Ashes	2
CULS_2014a	CULS_2014a_C	Pot	Wheat	Podebrady	AN_A-BioAsh_B	Synthetic N	BioAsh_B	Ashes	2
CULS_2014a	CULS_2014a_D	Pot	Wheat	Podebrady	AN_A-BioAsh_A	Synthetic N	BioAsh_A	Ashes	2
CULS_2014b	CULS_2014b_A	Pot	Maize	Humpolec_B	AN_A-BioAsh_B	Synthetic N	BioAsh_B	Ashes	2
CULS_2014b	CULS_2014b_B	Pot	Maize	Humpolec_B	AN_A-BioAsh_A	Synthetic N	BioAsh_A	Ashes	2
CULS_2014b	CULS_2014b_C	Pot	Maize	Podebrady	AN_A-BioAsh_B	Synthetic N	BioAsh_B	Ashes	2
CULS_2014b	CULS_2014b_D	Pot	Maize	Podebrady	AN_A-BioAsh_A	Synthetic N	BioAsh_A	Ashes	2
CULS_2014c	CULS_2014c_A	Field	Maize	Humpolec_C	CAN_B	Synthetic N	noP	Control	2
CULS_2014c	CULS_2014c_B	Field	Maize	Humpolec_C	CAN_B	Synthetic N	RP_C	Rock P	2
CULS_2014c	CULS_2014c_C	Field	Maize	Humpolec_C	CAN_B	Synthetic N	TSP_B	Soluble P	2

Experiment	Cluster	Type	Crop	Soil	Nitrogen		Phosphorous		no. of BE
Experiment	Cluster	Type	Crop	Soil	N source	N form	P source	P form	no. of BE
CULS_2014c	CULS_2014c_D	Field	Maize	Humpolec_C	CAN_B	Synthetic N	RP_C	Rock P	2
CULS_2014c	CULS_2014c_E	Field	Maize	Humpolec_C	CAN_B	Synthetic N	TSP_B	Soluble P	2
CULS_2014d	CULS_2014d_A	Field	Maize	Lukavec_A	CAN_B	Synthetic N	noP	Control	2
CULS_2014d	CULS_2014d_B	Field	Maize	Lukavec_A	CAN_B	Synthetic N	RP_C	Rock P	2
CULS_2014d	CULS_2014d_C	Field	Maize	Lukavec_A	CAN_B	Synthetic N	TSP_B	Soluble P	2
CULS_2014d	CULS_2014d_D	Field	Maize	Lukavec_A	CAN_B	Synthetic N	RP_C	Rock P	2
CULS_2014d	CULS_2014d_E	Field	Maize	Lukavec_A	CAN_B	Synthetic N	TSP_B	Soluble P	2
CULS_2014e	CULS_2014e_A	Pot	Maize	Humpolec_A	CalciumNitrate_A	Synthetic N	noP	Control	3
CULS_2014e	CULS_2014e_B	Pot	Maize	Humpolec_A	CalciumNitrate_A	Synthetic N	RP_C	Rock P	3
CULS_2014e	CULS_2014e_C	Pot	Maize	Humpolec_A	CalciumNitrate_A	Synthetic N	TSP_B	Soluble P	3
CULS_2014f	CULS_2014f_A	Pot	Maize	Lukavec_B	CalciumNitrate_A	Synthetic N	noP	Control	3
CULS_2014f	CULS_2014f_B	Pot	Maize	Lukavec_B	CalciumNitrate_A	Synthetic N	RP_C	Rock P	3
CULS_2014f	CULS_2014f_C	Pot	Maize	Lukavec_B	CalciumNitrate_A	Synthetic N	TSP_B	Soluble P	3
CULS_2015a	CULS_2015a_A	Pot	Wheat	Lhota	AN_A-PSS	Synthetic N	PSS	Biochar	1
CULS_2015a	CULS_2015a_B	Pot	Wheat	Lhota	AN_A-TSS-a	Mixture	TSS	Biochar	1
CULS_2015a	CULS_2015a_C	Pot	Wheat	Lhota	AN_A-SS_B-a	Synthetic N	SS_B	Sewage sludge	1
CULS_2015a	CULS_2015a_D	Pot	Wheat	Lhota	AN_A	Synthetic N	RP_C	Rock P	1
CULS_2015a	CULS_2015a_E	Pot	Wheat	Lhota	AN_A	Synthetic N	BioAsh_A	Ashes	1
CULS_2015a	CULS_2015a_F	Pot	Wheat	Lhota	AN_A	Synthetic N	SSA_A	Ashes	1
CULS_2015a	CULS_2015a_G	Pot	Wheat	Lhota	AN_A-BioChar_A	Synthetic N	BioChar_A	Biochar	1
CULS_2015a	CULS_2015a_H	Pot	Wheat	Lhota	AN_A	Synthetic N	TSP_B	Soluble P	1

Experiment	Cluster	Type	Crop	Soil	Nitrogen		Phosphorous		no. of BE
Experiment	Cluster	Type	Crop	Soil	N source	N form	P source	P form	no. of BE
CULS_2015b	CULS_2015b_A	Pot	Maize	Zamberk_A	AN_A	Synthetic N	noP	Control	11
CULS_2015b	CULS_2015b_B	Pot	Maize	Zamberk_A	AN_A-BioAsh_B	Synthetic N	BioAsh_B	Ashes	6
CULS_2015b	CULS_2015b_C	Pot	Maize	Zamberk_A	AN_A-BioAsh_A	Synthetic N	BioAsh_A	Ashes	10
CULS_2015b	CULS_2015b_D	Pot	Maize	Zamberk_A	AN_A-SS_B-b	Mixture	SS_B	Sewage sludge	10
CULS_2015b	CULS_2015b_E	Pot	Maize	Zamberk_A	AN_A-TSS-b	Mixture	TSS	Biochar	6
CULS_2015b	CULS_2015b_F	Pot	Maize	Zamberk_A	Dig_B	Organic N	Dig_B	Digestates	10
CULS_2015b	CULS_2015b_G	Pot	Maize	Zamberk_A	AN_A	Synthetic N	RP_C	Rock P	11
CULS_2015c	CULS_2015c_A	Field	Maize	Humpolec_D	CAN_B	Synthetic N	noP	Control	2
CULS_2015c	CULS_2015c_B	Field	Maize	Humpolec_D	UAN	Synthetic N	noP	Control	2
CULS_2015c	CULS_2015c_C	Field	Maize	Humpolec_D	CAN_B	Synthetic N	noP	Control	2
CULS_2015d	CULS_2015d_A	Pot	Tomato	Humpolec_A	AN_A	Synthetic N	noP	Control	4
CULS_2016a	CULS_2016a_A	Pot	Wheat	Lhota	AN_A	Synthetic N	PSS	Biochar	1
CULS_2016a	CULS_2016a_B	Pot	Wheat	Lhota	AN_A	Synthetic N	TSS	Biochar	1
CULS_2016a	CULS_2016a_C	Pot	Wheat	Lhota	AN_A	Synthetic N	SS_B	Sewage sludge	1
CULS_2016a	CULS_2016a_D	Pot	Wheat	Lhota	AN_A	Synthetic N	RP_C	Rock P	1
CULS_2016a	CULS_2016a_E	Pot	Wheat	Lhota	AN_A	Synthetic N	BioAsh_A	Ashes	1
CULS_2016a	CULS_2016a_F	Pot	Wheat	Lhota	AN_A	Synthetic N	SSA_A	Ashes	1
CULS_2016a	CULS_2016a_G	Pot	Wheat	Lhota	AN_A	Synthetic N	BioChar_A	Biochar	1
CULS_2016a	CULS_2016a_H	Pot	Wheat	Lhota	AN_A	Synthetic N	TSP_B	Soluble P	1
CULS_2016b	CULS_2016b_A	Pot	Maize	Zamberk_B	noN	Control	noP	Control	1
CULS_2016b	CULS_2016b_B	Pot	Maize	Zamberk_B	noN	Control	BioAsh_A	Ashes	1

Experiment	Cluster	Type	Crop	Soil	Nitrogen		Phosphorous		no. of BE
Experiment	Cluster	Type	Crop	Soil	N source	N form	P source	P form	no. of BE
CULS_2016b	CULS_2016b_C	Pot	Maize	Zamberk_B	AN_A	Synthetic N	noP	Control	1
CULS_2016b	CULS_2016b_D	Pot	Maize	Zamberk_B	AN_A	Synthetic N	BioAsh_A	Ashes	1
CULS_2016b	CULS_2016b_E	Pot	Maize	Zamberk_B	AN_A	Synthetic N	noP	Control	1
CULS_2016b	CULS_2016b_F	Pot	Maize	Zamberk_B	AN_A	Synthetic N	BioAsh_A	Ashes	1
CULS_2016b	CULS_2016b_G	Pot	Maize	Zamberk_B	AN_A	Synthetic N	noP	Control	1
CULS_2016b	CULS_2016b_H	Pot	Maize	Zamberk_B	AN_A	Synthetic N	BioAsh_A	Ashes	1
CULS_2016c	CULS_2016c_A	Pot	Maize	1	AN_A	Synthetic N	noP	Control	2
CULS_2016c	CULS_2016c_B	Pot	Maize	101	AN_A	Synthetic N	noP	Control	2
CULS_2016c	CULS_2016c_C	Pot	Maize	2	AN_A	Synthetic N	noP	Control	2
CULS_2016c	CULS_2016c_D	Pot	Maize	14	AN_A	Synthetic N	noP	Control	2
CULS_2016d	CULS_2016d_A	Field	Maize	Humpolec_E	CalciumNitrate_A	Synthetic N	RP_E	Rock P	3
CULS_2016d	CULS_2016d_B	Field	Maize	Humpolec_E	NovaTec	Ammonium	RP_E	Rock P	3
CULS_2016e	CULS_2016e_A	Field	Maize	Lipa	CalciumNitrate_A	Synthetic N	RP_E	Rock P	3
CULS_2016e	CULS_2016e_B	Field	Maize	Lipa	NovaTec	Ammonium	RP_E	Rock P	3
CULS_2017a	CULS_2017a_A	Pot	Wheat	Citov	AN_A	Synthetic N	-	NA	2
CULS_2017a	CULS_2017a_B	Pot	Wheat	Citov	AN_A	Synthetic N	RP_D	Rock P	2
CULS_2017a	CULS_2017a_C	Pot	Wheat	Citov	AN_A	Synthetic N	TSP_B	Soluble P	2
FiBL_2013a	FiBL_2013a_A	Pot	Maize	Buus	CAN_A	Synthetic N	RP_A	Soluble P	3
FiBL_2013a	FiBL_2013a_B	Pot	Maize	DOK-M	CAN_A	Synthetic N	RP_A	Soluble P	3
FiBL_2013a	FiBL_2013a_C	Pot	Maize	Le-Caron	CAN_A	Synthetic N	RP_A	Soluble P	3
FiBL_2014a	FiBL_2014a_A	Pot	Maize	Buus	CAN_A	Synthetic N	noP	Control	4

Experiment	Cluster	Type	Crop	Soil	Nitrogen		Phosphorous		no. of BE
Experiment	Cluster	Type	Crop	Soil	N source	N form	P source	P form	no. of BE
FiBL_2014a	FiBL_2014a_B	Pot	Maize	Buus	CAN_A	Synthetic N	TSP_A	Soluble P	4
FiBL_2014a	FiBL_2014a_C	Pot	Maize	Buus	CAN_A	Synthetic N	RP_B	Rock P	4
FiBL_2014a	FiBL_2014a_D	Pot	Maize	Buus	CAN_A-Dig_A	Organic N	Dig_A	Digestates	4
FiBL_2014a	FiBL_2014a_E	Pot	Maize	Buus	CAN_A-Comp_B	Synthetic N	Comp_B	Compost	4
FiBL_2014b	FiBL_2014b_A	Field	Maize	Buus	Hornmeal	Organic N	Comp_B	Compost	2
FiBL_2014b	FiBL_2014b_B	Field	Maize	Buus	Hornmeal	Organic N	Comp_B	Compost	2
FiBL_2014c	FiBL_2014c_A	Field	Maize	Hagenwil	Man_B	Organic N	Man_C	Manure	2
FiBL_2015a	FiBL_2015a_A	Pot	Maize	Buus	CAN_A	Synthetic N	noP	Control	5
FiBL_2015a	FiBL_2015a_B	Pot	Maize	Buus	CAN_A	Synthetic N	RP_C	Rock P	5
FiBL_2015a	FiBL_2015a_C	Pot	Maize	Buus	CAN_A-Comp_B	Synthetic N	Comp_B	Compost	5
FiBL_2015a	FiBL_2015a_D	Pot	Maize	Buus	CAN_A-Dig_A	Organic N	Dig_A	Digestates	5
FiBL_2015a	FiBL_2015a_E	Pot	Maize	Buus	CAN_A-CompMan_B	Mixture	CompMan_B	Manure	5
FiBL_2015a	FiBL_2015a_F	Pot	Maize	Buus	CAN_A-CompMan_A	Mixture	CompMan_A	Manure	5
FiBL_2015b	FiBL_2015b_A	Pot	Maize	Dompierre	CAN_A	Synthetic N	noP	Control	5
FiBL_2015b	FiBL_2015b_B	Pot	Maize	Dompierre	CAN_A	Synthetic N	RP_C	Rock P	5
FiBL_2015b	FiBL_2015b_C	Pot	Maize	Dompierre	CAN_A-Comp_B	Synthetic N	Comp_B	Compost	5
FiBL_2015b	FiBL_2015b_D	Pot	Maize	Dompierre	CAN_A-Dig_A	Organic N	Dig_A	Digestates	5
FiBL_2015b	FiBL_2015b_E	Pot	Maize	Dompierre	CAN_A-CompMan_B	Mixture	CompMan_B	Manure	5
FiBL_2015b	FiBL_2015b_F	Pot	Maize	Dompierre	CAN_A-CompMan_A	Mixture	CompMan_A	Manure	5
FiBL_2015c	FiBL_2015c_A	Pot	Maize	Buus	CAN_A	Synthetic N	noP	Control	3
FiBL_2015c	FiBL_2015c_B	Pot	Maize	Buus	CAN_A-CompMan_A	Mixture	CompMan_A	Manure	3

Experiment	Cluster	Type	Crop	Soil	Nitrogen		Phosphorous		no. of BE
Experiment	Cluster	Type	Crop	Soil	N source	N form	P source	P form	no. of BE
FiBL_2015d	FiBL_2015d_A	Field	Maize	Buus	Hornmeal	Organic N	noP	Control	2
FiBL_2015d	FiBL_2015d_B	Field	Maize	Buus	Hornmeal-Comp_B	Organic N	Comp_B	Compost	2
FiBL_2015e	FiBL_2015e_A	Field	Maize	Buus	Hornmeal	Organic N	noP	Control	2
FiBL_2015e	FiBL_2015e_B	Field	Maize	Buus	CompMan_A	Organic N	CompMan_A	Manure	3
FiBL_2015f	FiBL_2015f_A	Field	Maize	Hagenwil	Man_B	Organic N	noP	Control	2
FiBL_2016a	FiBL_2016a_A	Pot	Maize	Dompierre	CAN_A	Synthetic N	noP	Control	1
FiBL_2016a	FiBL_2016a_B	Pot	Maize	Dompierre	CAN_A	Synthetic N	RP_E	Rock P	1
FiBL_2016a	FiBL_2016a_C	Pot	Maize	Dompierre	Comp_B	Organic N	Comp_B	Compost	1
FiBL_2016a	FiBL_2016a_D	Pot	Maize	Dompierre	CompMan_A	Organic N	CompMan_A	Manure	1
FiBL_2016a	FiBL_2016a_E	Pot	Maize	Dompierre	CAN_A	Synthetic N	SSA_B	Ashes	1
HKKALKE_2013a	HKKALKE_2013a_A	Pot	Wheat	Voerden	CalciumNitrate_C	Synthetic N	noP	Control	3
HKKALKE_2013a	HKKALKE_2013a_B	Pot	Wheat	Voerden	CalciumNitrate_C	Synthetic N	TSP_C	Soluble P	3
HKKALKE_2013a	HKKALKE_2013a_C	Pot	Wheat	Voerden	CalciumNitrate_C	Synthetic N	RP_A	Soluble P	3
HKKALKE_2013a	HKKALKE_2013a_D	Pot	Wheat	Voerden	CalciumNitrate_C-SS_C	Synthetic N	SS_C	Sewage sludge	3
HKKALKE_2013a	HKKALKE_2013a_E	Pot	Wheat	Voerden	CalciumNitrate_C	Synthetic N	SSA_C	Ashes	3
HKKALKE_2013a	HKKALKE_2013a_F	Pot	Wheat	Voerden	CalciumNitrate_C	Synthetic N	TP	Soluble P	3
HKKALKE_2013a	HKKALKE_2013a_G	Pot	Wheat	Voerden	CalciumNitrate_C	Synthetic N	SSA-BOFS	Soluble P	3
HKKALKE_2014a	HKKALKE_2014a_A	Pot	Maize	Voerden	CalciumNitrate_C	Synthetic N	noP	Control	3
HKKALKE_2014a	HKKALKE_2014a_B	Pot	Maize	Voerden	CalciumNitrate_C	Synthetic N	TSP_C	Soluble P	3
HKKALKE_2014a	HKKALKE_2014a_C	Pot	Maize	Voerden	CalciumNitrate_C	Synthetic N	RP_C	Rock P	3
HKKALKE_2014a	HKKALKE_2014a_D	Pot	Maize	Voerden	CalciumNitrate_C-GSS	Synthetic N	GSS	Sewage sludge	3

Experiment	Cluster	Type	Crop	Soil	Nitrogen		Phosphorous		no. of BE
Experiment	Cluster	Type	Crop	Soil	N source	N form	P source	P form	no. of BE
HKKALKE_2014a	HKKALKE_2014a_E	Pot	Maize	Voerden	CalciumNitrate_C	Synthetic N	SSA_C	Ashes	3
HKKALKE_2014a	HKKALKE_2014a_F	Pot	Maize	Voerden	CalciumNitrate_C-Comp_C	Mixture	Comp_C	Compost	3
HKKALKE_2014a	HKKALKE_2014a_G	Pot	Maize	Voerden	CalciumNitrate_C	Synthetic N	SSA-BOFS	Soluble P	3
HKKALKE_2015a	HKKALKE_2015a_A	Pot	Maize	Voerden	AmmoniumSulphate	Ammonium	noP	Control	2
HKKALKE_2015a	HKKALKE_2015a_B	Pot	Maize	Voerden	AmmoniumSulphate	Ammonium	TSP_C	Soluble P	2
HKKALKE_2015a	HKKALKE_2015a_C	Pot	Maize	Voerden	AmmoniumSulphate	Ammonium	RP_C	Rock P	2
HKKALKE_2015a	HKKALKE_2015a_D	Pot	Maize	Voerden	AmmoniumSulphate-Dig_C	Synthetic N	Dig_C	Digestates	2
HKKALKE_2015a	HKKALKE_2015a_E	Pot	Maize	Voerden	AmmoniumSulphate	Ammonium	SSA_C	Ashes	2
HKKALKE_2015a	HKKALKE_2015a_F	Pot	Maize	Voerden	AmmoniumSulphate	Ammonium	SSA-BOFS	Soluble P	2
JKI_2013a	JKI_2013a_A	Pot	Tomato	UHOH campus - A	CalciumNitrate_B	Synthetic N	CalciumdiHP	Soluble P	4
JKI_2013b	JKI_2013b_A	Pot	Maize	UHOH campus - A	CalciumNitrate_B	Synthetic N	CalciumdiHP	Soluble P	4
JKI_2014a	JKI_2014a_A	Pot	Tomato	Karlsruhe_A	CalciumNitrate_B	Synthetic N	CalciumdiHP	Soluble P	3
JKI_2014b	JKI_2014b_A	Pot	Maize	Karlsruhe_A	CalciumNitrate_B	Synthetic N	CalciumdiHP	Soluble P	3
JKI_2016a	JKI_2016a_A	Pot	Maize	Sanitz_A	noN	Control	noP	Control	1
JKI_2016a	JKI_2016a_B	Pot	Maize	Bayern	noN	Control	noP	Control	1
UCPH_2013a	UCPH_2013a_A	Pot	Wheat	NDT-A	CAN_A	Synthetic N	noP	Control	3
UCPH_2013a	UCPH_2013a_B	Pot	Wheat	NDT-A	CAN_A	Synthetic N	TSP_A	Soluble P	3
UCPH_2013a	UCPH_2013a_C	Pot	Wheat	NDT-A	CAN_A	Synthetic N	RP_A	Soluble P	3
UCPH_2013a	UCPH_2013a_D	Pot	Wheat	NDT-A	CAN_A-SS_A	Synthetic N	SS_A	Sewage sludge	3
UCPH_2013a	UCPH_2013a_E	Pot	Wheat	NDT-A	CAN_A	Synthetic N	SSA_A	Ashes	3
UCPH_2013a	UCPH_2013a_F	Pot	Wheat	NDT-A	CAN_A-Man_A	Synthetic N	Man_A	Manure	3

Experiment	Cluster	Type	Crop	Soil	Nitrogen		Phosphorous		no. of BE
Experiment	Cluster	Type	Crop	Soil	N source	N form	P source	P form	no. of BE
UCPH_2013a	UCPH_2013a_G	Pot	Wheat	NDT-A	CAN_A-Comp_A	Mixture	Comp_A	Compost	3
UCPH_2013b	UCPH_2013b_A	Pot	Wheat	NDT-A	CAN_A-SS_A	Synthetic N	SS_A	Sewage sludge	3
UCPH_2013b	UCPH_2013b_B	Pot	Wheat	NDT-A	CAN_A	Synthetic N	SSA_A	Ashes	3
UCPH_2013b	UCPH_2013b_C	Pot	Wheat	NDT-A	CAN_A-Comp_A	Mixture	Comp_A	Compost	3
UCPH_2013b	UCPH_2013b_D	Pot	Wheat	NDT-E	CAN_A	Synthetic N	noP	Control	3
UCPH_2013b	UCPH_2013b_E	Pot	Wheat	NDT-E	CAN_A	Synthetic N	SSA_A	Ashes	3
UCPH_2014a	UCPH_2014a_A	Pot	Maize	NDT-A	CAN_A	Synthetic N	noP	Control	7
UCPH_2014a	UCPH_2014a_B	Pot	Maize	NDT-A	CAN_A	Synthetic N	SSA_A	Ashes	7
UCPH_2014a	UCPH_2014a_C	Pot	Maize	NDT-A	CAN_A-SS_A	Synthetic N	SS_A	Sewage sludge	7
UCPH_2014a	UCPH_2014a_D	Pot	Maize	CRUCIAL-U_A	CAN_A	Synthetic N	noP	Control	7
UCPH_2015a	UCPH_2015a_A	Pot	Maize	CRUCIAL-U_B	Solution_A	Synthetic N	noP	Control	4
UCPH_2015a	UCPH_2015a_B	Pot	Maize	CRUCIAL-U_B	Solution_A	Synthetic N	noP	Control	4
UCPH_2015a	UCPH_2015a_C	Pot	Maize	CRUCIAL-CMA	Solution_A	Synthetic N	noP	Control	4
UCPH_2015a	UCPH_2015a_D	Pot	Maize	CRUCIAL-CMA	Solution_A	Synthetic N	noP	Control	4
UHOHa_2013a	UHOHa_2013a_A	Pot	Maize	C-Loess_A	CalciumNitrate_C	Synthetic N	noP	Control	9
UHOHa_2013b	UHOHa_2013b_A	Pot	Tomato	JR	CalciumNitrate_C	Synthetic N	CalciumdiHP	Soluble P	3
UHOHa_2013b	UHOHa_2013b_B	Pot	Tomato	JR + Manure	Man_D	Organic N	Man_D	Manure	3
UHOHa_2013c	UHOHa_2013c_A	Pot	Maize	UHOH vineyard	CalciumNitrate_C	Synthetic N	noP	Control	4
UHOHa_2014a	UHOHa_2014a_A	Pot	Maize	Kleinhohenheim_D	CalciumNitrate_C	Synthetic N	CalciumdiHP	Soluble P	7
UHOHa_2014b	UHOHa_2014b_A	Pot	Maize	UHOH vineyard	CalciumNitrate_C	Synthetic N	CalciumdiHP	Soluble P	4
UHOHa_2014c	UHOHa_2014c_A	Pot	Tomato	UHOH vineyard	CalciumNitrate_C	Synthetic N	CalciumdiHP	Soluble P	4

Experiment	Cluster	Type	Crop	Soil	Nitrogen		Phosphorous		no. of BE
Experiment	Cluster	Type	Crop	Soil	N source	N form	P source	P form	no. of BE
UHOHa_2014d	UHOHa_2014d_A	Pot	Tomato	Karlsruhe_B	CalciumNitrate_C	Synthetic N	CalciumdiHP	Soluble P	3
UHOHa_2014d	UHOHa_2014d_B	Pot	Tomato	Karlsruhe_B_sterilized	CalciumNitrate_C	Synthetic N	CalciumdiHP	Soluble P	3
UHOHa_2014e	UHOHa_2014e_A	Pot	Tomato	Karlsruhe_B	CalciumNitrate_C	Synthetic N	CalciumdiHP	Soluble P	2
UHOHa_2014e	UHOHa_2014e_B	Pot	Tomato	Karlsruhe_B	CalciumNitrate_C	Synthetic N	CalciumdiHP	Soluble P	2
UHOHa_2014e	UHOHa_2014e_C	Pot	Tomato	Karlsruhe_B	CalciumNitrate_C	Synthetic N	CalciumdiHP	Soluble P	2
UHOHa_2014f	UHOHa_2014f_A	Pot	Maize	IHOF_E	CalciumNitrate_B	Synthetic N	CalciumdiHP	Soluble P	10
UHOHa_2014g	UHOHa_2014g_A	Pot	Maize	IHOF_F	Duratec	Ammonium	Duratec	Soluble P	4
UHOHa_2014g	UHOHa_2014g_A	Pot	Maize	IHOF_F	CalciumNitrate_B	Synthetic N	CalciumdiHP	Soluble P	4
UHOHa_2014h	UHOHa_2014h_A	Pot	Maize	Baven_B+Kleinh_D	CalciumNitrate_C	Synthetic N	MAP	Soluble P	5
UHOHa_2014i	UHOHa_2014i_A	Pot	Maize	Karlsruhe_B	CalciumNitrate_C	Synthetic N	CalciumdiHP	Soluble P	2
UHOHa_2014i	UHOHa_2014i_B	Pot	Maize	Karlsruhe_B	CalciumNitrate_C	Synthetic N	CalciumdiHP	Soluble P	2
UHOHa_2014i	UHOHa_2014i_C	Pot	Maize	Karlsruhe_B	CalciumNitrate_C	Synthetic N	CalciumdiHP	Soluble P	2
UHOHa_2014j	UHOHa_2014j_A	Field	Maize	IHOF_A	NovaTec	Ammonium	MAP	Soluble P	4
UHOHa_2014k	UHOHa_2014k_A	Field	Maize	IHOF_A	NovaTec	Ammonium	MAP	Soluble P	3
UHOHa_2014l	UHOHa_2014l_A	Pot	Maize	IHOF_C	CalciumNitrate_B	Synthetic N	CalciumdiHP	Soluble P	3
UHOHa_2015a	UHOHa_2015a_A	Pot	Maize	Karlsruhe_C	CalciumNitrate_C	Synthetic N	CalciumdiHP	Soluble P	2
UHOHa_2015b	UHOHa_2015b_A	Field	Maize	IHOF_B	CompMan_A	Organic N	CompMan_A	Manure	2
UHOHa_2015b	UHOHa_2015b_B	Field	Maize	IHOF_B	HAST	Synthetic N	noP	Control	2
UHOHa_2015b	UHOHa_2015b_C	Field	Maize	IHOF_B	NovaTec	Ammonium	DAP	Soluble P	2
UHOHa_2015c	UHOHa_2015c_A	Field	Maize	IHOF_B	NovaTec	Ammonium	DAP	Soluble P	3
UHOHa_2015d	UHOHa_2015d_A	Pot	Maize	Kleinhohenheim_E	CalciumNitrate_C	Synthetic N	CalciumdiHP	Soluble P	1

Experiment	Cluster	Type	Crop	Soil	Nitrogen		Phosphorous		no. of BE
Experiment	Cluster	Type	Crop	Soil	N source	N form	P source	P form	no. of BE
UHOHa_2015d	UHOHa_2015d_B	Pot	Maize	Kleinhohenheim_E	CalciumNitrate_C	Synthetic N	CalciumdiHP	Soluble P	1
UHOHa_2015e	UHOHa_2015e_A	Pot	Maize	Kleinhohenheim_E	CalciumNitrate_C	Synthetic N	CalciumdiHP	Soluble P	1
UHOHa_2015g	UHOHa_2015g_A	Pot	Maize	Kleinhohenheim_F	Comp_D	Organic N	Comp_D	Compost	1
UHOHa_2015g	UHOHa_2015g_B	Pot	Maize	Kleinhohenheim_F	SS_D	Organic N	SS_D	Sewage sludge	1
UHOHa_2015g	UHOHa_2015g_C	Pot	Maize	Kleinhohenheim_F	CompMan_A	Organic N	CompMan_A	Manure	1
UHOHa_2015h	UHOHa_2015h_A	Pot	Maize	Kleinhohenheim_F	MinN	Synthetic N	noP	Control	1
UHOHa_2015h	UHOHa_2015h_B	Pot	Maize	Kleinhohenheim_F	MinA	Ammonium	noP	Control	1
UHOHa_2015h	UHOHa_2015h_C	Pot	Maize	Kleinhohenheim_F	CompMan_A	Organic N	CompMan_A	Manure	1
UHOHa_2015h	UHOHa_2015h_D	Pot	Maize	Kleinhohenheim_F	CompMan_C	Organic N	CompMan_C	Manure	1
UHOHa_2015h	UHOHa_2015h_E	Pot	Maize	Kleinhohenheim_F	CompMan_A-DMPP	Organic N	CompMan_A-DMPP	Manure	1
UHOHa_2015h	UHOHa_2015h_F	Pot	Maize	Kleinhohenheim_F	CompMan_C-DMPP	Organic N	CompMan_C-DMPP	Manure	1
UHOHa_2015i	UHOHa_2015i_A	Pot	Tomato	Kleinhohenheim_F	Duratec	Ammonium	Duratec	Soluble P	3
UHOHa_2015i	UHOHa_2015i_B	Pot	Tomato	Kleinhohenheim_F	CompMan_C	Organic N	CompMan_C	Manure	3
UHOHa_2016a	UHOHa_2016a_A	Pot	Maize	Karlsruhe_C	CalciumNitrate_C	Synthetic N	CalciumdiHP	Soluble P	1
UHOHa_2016c	UHOHa_2016c_A	Pot	Maize	IHOF_D	NovaTec	Ammonium	CalciumdiHP	Soluble P	4
UHOHa_2016d	UHOHa_2016d_A	Pot	Maize	IHOF_D	CAN_A	Synthetic N	CalciumdiHP	Soluble P	4
UHOHa_2016e	UHOHa_2016e_A	Pot	Maize	IHOF_D	NovaTec	Ammonium	CalciumdiHP	Soluble P	3
UHOHa_2016f	UHOHa_2016f_A	Pot	Maize	IHOF_D	CAN_A	Synthetic N	CalciumdiHP	Soluble P	3
UHOHa_2016g	UHOHa_2016g_A	Pot	Maize	HeiHo	NovaTec	Ammonium	CalciumdiHP	Soluble P	3
UHOHa_2016h	UHOHa_2016h_A	Pot	Maize	HeiHo	CAN_A	Synthetic N	CalciumdiHP	Soluble P	3
UHOHa_2016i	UHOHa_2016i_A	Pot	Maize	HeiHo	NovaTec	Ammonium	CalciumdiHP	Soluble P	3

Experiment	Cluster	Type	Crop	Soil	Nitrogen		Phosphorous		no. of BE
Experiment	Cluster	Type	Crop	Soil	N source	N form	P source	P form	no. of BE
UHOHa_2016j	UHOHa_2016j_A	Pot	Maize	HeiHo	CAN_A	Synthetic N	CalciumdiHP	Soluble P	3
UHOHa_2016k	UHOHa_2016k_A	Field	Maize	IHOF_D	NovaTec	Ammonium	DAP	Soluble P	10
UHOHa_2016l	UHOHa_2016l_A	Field	Wheat	Horb_A	ASN_CAN	Ammonium	noP	Control	6
UHOHa_2016m	UHOHa_2016m_A	Pot	Maize	Horb_B	CAN_A	Synthetic N	CalciumdiHP	Soluble P	1
UHOHa_2016m	UHOHa_2016m_B	Pot	Maize	Horb_B	NovaTec	Ammonium	CalciumdiHP	Soluble P	1
UHOHa_2016m	UHOHa_2016m_C	Pot	Maize	Horb_B	CAN_A	Synthetic N	CalciumdiHP	Soluble P	1
UHOHa_2016m	UHOHa_2016m_D	Pot	Maize	Horb_B	NovaTec	Ammonium	CalciumdiHP	Soluble P	1
UHOHa_2016n	UHOHa_2016n_A	Pot	Maize	Horb_C	CalciumNitrate_B	Synthetic N	CalciumdiHP	Soluble P	2
UHOHa_2016n	UHOHa_2016n_B	Pot	Maize	Horb_C	CalciumNitrate_B	Synthetic N	CalciumdiHP	Soluble P	2
UHOHa_2016o	UHOHa_2016o_A	Field	Maize	IHOF_F	NovaTec-DAP	Synthetic N	DAP	Soluble P	1
UHOHb_2013a	UHOHb_2013a_A	Pot	Maize	Kleinhohenheim_A	CalciumNitrate_B	Synthetic N	noP	Control	4
UHOHb_2013a	UHOHb_2013a_B	Pot	Maize	Kleinhohenheim_A	CalciumNitrate_B	Synthetic N	noP	Control	13
UHOHb_2013b	UHOHb_2013b_A	Pot	Maize	Kleinhohenheim_A	CalciumNitrate_B	Synthetic N	SSA_C	Ashes	1
UHOHb_2013b	UHOHb_2013b_B	Pot	Maize	Kleinhohenheim_A	CalciumNitrate_B	Synthetic N	SSA_C	Ashes	1
UHOHb_2014a	UHOHb_2014a_A	Pot	Maize	Weinberg	AS-Man_D	Mixture	noP	Control	1
UHOHb_2014a	UHOHb_2014a_B	Pot	Maize	Weinberg	AS-Man_D	Mixture	RP_D-Man_D	Rock P	1
UHOHb_2014a	UHOHb_2014a_C	Pot	Maize	Weinberg	CaN-Man_D	Mixture	RP_D-Man_D	Rock P	1
UHOHb_2014a	UHOHb_2014a_D	Pot	Maize	Weinberg	AS-Man_D	Mixture	RP_D-Man_D	Rock P	1
UHOHb_2014b	UHOHb_2014b_A	Pot	Maize	C-Loess_B	CalciumNitrate_B	Synthetic N	RP_D	Rock P	4
UHOHb_2014c	UHOHb_2014c_A	Pot	Maize	C-Loess_B	CalciumNitrate_B	Synthetic N	RP_D	Rock P	4
UHOHb_2014d	UHOHb_2014d_A	Pot	Maize	C-Loess_B	CalciumNitrate_B	Synthetic N	RP_D	Rock P	4

Experiment	Cluster	Type	Crop	Soil	Nitrogen		Phosphorous		no. of BE
**Experiment**	**Cluster**	**Type**	**Crop**	**Soil**	**N source**	**N form**	**P source**	**P form**	**no. of BE**
UHOHb_2014e	UHOHb_2014e_A	Pot	Maize	C-Loess_B	CalciumNitrate_B	Synthetic N	CalciumdiHP	Soluble P	1
UHOHb_2014e	UHOHb_2014e_B	Pot	Maize	C-Loess_B	AmmoniumSulphate	Ammonium	CalciumdiHP	Soluble P	1
UHOHb_2014f	UHOHb_2014f_A	Field	Maize	IHOF_A	NovaTec	Ammonium	EasyStartE4	Soluble P	1
UHOHb_2014f	UHOHb_2014f_B	Field	Maize	IHOF_A	NovaTec	Ammonium	MAP	Soluble P	2
UHOHb_2014f	UHOHb_2014f_C	Field	Maize	IHOF_A	NovaTec	Ammonium	MAP	Soluble P	2
UHOHb_2014f	UHOHb_2014f_D	Field	Maize	IHOF_A	NovaTec	Ammonium	MAP	Soluble P	2
UHOHb_2014g	UHOHb_2014g_A	Pot	Maize	Weinberg	noN	Control	noP	Control	1
UHOHb_2014g	UHOHb_2014g_B	Pot	Maize	Weinberg	noN	Control	noP	Control	1
UHOHb_2014g	UHOHb_2014g_C	Pot	Maize	Weinberg	CalciumNitrate_B	Synthetic N	noP	Control	1
UHOHb_2014g	UHOHb_2014g_D	Pot	Maize	Weinberg	CalciumNitrate_B	Synthetic N	noP	Control	1
UHOHb_2014g	UHOHb_2014g_E	Pot	Maize	Weinberg	AmmoniumSulphate	Ammonium	noP	Control	1
UHOHb_2014g	UHOHb_2014g_F	Pot	Maize	Weinberg	AmmoniumSulphate	Ammonium	noP	Control	1
UHOHb_2014g	UHOHb_2014g_G	Pot	Maize	Weinberg	AmmoniumSulphate	Ammonium	noP	Control	1
UHOHb_2015a	UHOHb_2015a_A	Pot	Maize	Low P-sorbing Soil	CalciumNitrate_B	Synthetic N	RP_D	Rock P	1
UHOHb_2015a	UHOHb_2015a_B	Pot	Maize	High P-sorbing Soil	CalciumNitrate_B	Synthetic N	RP_D	Rock P	1
UHOHb_2015a	UHOHb_2015a_C	Pot	Maize	Low P-sorbing Soil	AmmoniumSulphate	Ammonium	RP_D	Rock P	1
UHOHb_2015a	UHOHb_2015a_D	Pot	Maize	High P-sorbing Soil	AmmoniumSulphate	Ammonium	RP_D	Rock P	1
UHOHb_2015b	UHOHb_2015b_A	Pot	Maize	Kleinhohenheim_B	AmmoniumSulphate	Ammonium	RP_D	Rock P	7
UHOHb_2015c	UHOHb_2015c_A	Pot	Wheat	Barvendorf_A	CalciumNitrate_B	Synthetic N	RP_D	Rock P	1
UHOHb_2015c	UHOHb_2015c_B	Pot	Wheat	Barvendorf_A	AmmoniumSulphate	Ammonium	RP_D	Rock P	4
UHOHb_2015d	UHOHb_2015d_A	Field	Maize	IHOF_B	NovaTec	Ammonium	DAP	Soluble P	2

Experiment	Cluster	Type	Crop	Soil	Nitrogen		Phosphorous		no. of BE
Experiment	Cluster	Type	Crop	Soil	N source	N form	P source	P form	no. of BE
UHOHb_2015d	UHOHb_2015d_B	Field	Maize	IHOF_B	NovaTec	Ammonium	DAP	Soluble P	2
UHOHb_2015e	UHOHb_2015e_A	Pot	Maize	Kleinhohenheim_G	NovaTec	Ammonium	noP	Control	2
UHOHb_2015e	UHOHb_2015e_B	Pot	Maize	Kleinhohenheim_G	NovaTec	Ammonium	RP_D	Rock P	2
UHOHb_2015e	UHOHb_2015e_C	Pot	Maize	Kleinhohenheim_G	NovaTec	Ammonium	Struvite	Soluble P	2
UHOHb_2015e	UHOHb_2015e_D	Pot	Maize	Kleinhohenheim_G	NovaTec	Ammonium	SSA_C	Ashes	2
UHOHc_2014a	UHOHc_2014a_A	Pot	Tomato	UHOH campus	CalciumNitrate_B	Synthetic N	CalciumdiHP	Soluble P	2
UHOHc_2014b	UHOHc_2014b_A	Pot	Maize	UHOH campus	CalciumNitrate_B	Synthetic N	CalciumdiHP	Soluble P	2
UHOHc_2016	UHOHc_2016_A	Pot	Tomato	UHOH campus	CalciumNitrate_B	Synthetic N	CalciumdiHP	Soluble P	2
UHOHc_2016	UHOHc_2016_B	Pot	Tomato	UHOH campus	CalciumNitrate_B	Synthetic N	CalciumdiHP	Soluble P	2
UNINAa_2013a	UNINAa_2013a_A	Pot	Tomato	peat moss_UNINAa	Solution_B	Synthetic N	Solution_B	Soluble P	4
UNINAa_2013a	UNINAa_2013a_B	Pot	Tomato	peat moss_UNINAa	Solution_B	Synthetic N	Solution_B	Soluble P	4
UNINAa_2013a	UNINAa_2013a_C	Pot	Tomato	peat moss_UNINAa	Solution_B	Synthetic N	Solution_B	Soluble P	4
UNINAa_2013a	UNINAa_2013a_D	Pot	Tomato	peat moss_UNINAa	Solution_C	Synthetic N	Solution_C	Soluble P	4
UNINAa_2013a	UNINAa_2013a_E	Pot	Tomato	peat moss_UNINAa	Solution_C	Synthetic N	Solution_C	Soluble P	4
UNINAa_2013a	UNINAa_2013a_F	Pot	Tomato	peat moss_UNINAa	Solution_C	Synthetic N	Solution_C	Soluble P	4
UNINAa_2013a	UNINAa_2013a_G	Pot	Tomato	peat moss_UNINAa	Solution_D	Synthetic N	Solution_D	Soluble P	4
UNINAa_2013a	UNINAa_2013a_H	Pot	Tomato	peat moss_UNINAa	Solution_D	Synthetic N	Solution_D	Soluble P	4
UNINAa_2013a	UNINAa_2013a_I	Pot	Tomato	peat moss_UNINAa	Solution_D	Synthetic N	Solution_D	Soluble P	4
UNINAa_2014a	UNINAa_2014a_A	Pot	Tomato	peat moss_UNINAa	Solution_E	Synthetic N	Solution_E	Soluble P	2
UNINAa_2014a	UNINAa_2014a_B	Pot	Tomato	peat moss_UNINAa	Solution_E	Synthetic N	Solution_E	Soluble P	2
UNINAa_2014a	UNINAa_2014a_C	Pot	Tomato	peat moss_UNINAa	Solution_E	Synthetic N	Solution_E	Soluble P	2

Experiment	Cluster	Type	Crop	Soil	Nitrogen		Phosphorous		no. of BE
Experiment	Cluster	Type	Crop	Soil	N source	N form	P source	P form	no. of BE
UNINAa_2014a	UNINAa_2014a_D	Pot	Tomato	peat moss_UNINAa	Solution_F	Synthetic N	Solution_F	Soluble P	2
UNINAa_2014a	UNINAa_2014a_E	Pot	Tomato	peat moss_UNINAa	Solution_F	Synthetic N	Solution_F	Soluble P	2
UNINAa_2014a	UNINAa_2014a_F	Pot	Tomato	peat moss_UNINAa	Solution_F	Synthetic N	Solution_F	Soluble P	2
UNINAa_2015a	UNINAa_2015a_A	Pot	Tomato	peat moss_UNINAa	Solution_G	Synthetic N	Solution_G	Soluble P	1
UNINAa_2015a	UNINAa_2015a_B	Pot	Tomato	peat moss_UNINAa	Solution_G	Synthetic N	Solution_G	Soluble P	1
UNINAa_2015a	UNINAa_2015a_C	Pot	Tomato	peat moss_UNINAa	Solution_G	Synthetic N	Solution_G	Soluble P	1
UNINAa_2015a	UNINAa_2015a_D	Pot	Tomato	peat moss_UNINAa	Solution_H	Synthetic N	Solution_H	Soluble P	1
UNINAa_2015a	UNINAa_2015a_E	Pot	Tomato	peat moss_UNINAa	Solution_H	Synthetic N	Solution_H	Soluble P	1
UNINAa_2015a	UNINAa_2015a_F	Pot	Tomato	peat moss_UNINAa	Solution_H	Synthetic N	Solution_H	Soluble P	1
UNINAa_2015b	UNINAa_2015b_A	Field	Tomato	UNINAa soil_A	Solution_E	Synthetic N	Solution_E	Soluble P	2
UNINAa_2015b	UNINAa_2015b_B	Field	Tomato	UNINAa soil_A	Solution_G	Synthetic N	Solution_G	Soluble P	2
UNINAa_2015b	UNINAa_2015b_C	Field	Tomato	UNINAa soil_A	Solution_E	Synthetic N	Solution_E	Soluble P	2
UNINAa_2015b	UNINAa_2015b_D	Field	Tomato	UNINAa soil_A	Solution_G	Synthetic N	Solution_G	Soluble P	2
UNINAa_2016a	UNINAa_2016a_A	Field	Wheat	UNINAa soil_B	HAST-NP_B	Synthetic N	NP_B	Soluble P	2
UNINAa_2016a	UNINAa_2016a_B	Field	Wheat	UNINAa soil_B	HAST-NP_B	Synthetic N	NP_B	Soluble P	2
UNINAa_2016b	UNINAa_2016b_A	Pot	Wheat	peat moss_UNINAa	AN_B	Synthetic N	noP	Control	2
UNINAa_2016b	UNINAa_2016b_B	Pot	Wheat	peat moss_UNINAa	AN_B	Synthetic N	noP	Control	2
UNINAa_2016b	UNINAa_2016b_C	Pot	Wheat	peat moss_UNINAa	AN_B	Synthetic N	noP	Control	2
UNINAa_2016b	UNINAa_2016b_D	Pot	Wheat	peat moss_UNINAa	noN	Control	noP	Control	2
UNINAa_2016b	UNINAa_2016b_E	Pot	Wheat	peat moss_UNINAa	noN	Control	noP	Control	2
UNINAa_2016b	UNINAa_2016b_F	Pot	Wheat	peat moss_UNINAa	noN	Control	noP	Control	2

Experiment	Cluster	Type	Crop	Soil	Nitrogen		Phosphorous		no. of BE
Experiment	Cluster	Type	Crop	Soil	N source	N form	P source	P form	no. of BE
UNINAb_2013a	UNINAb_2013a_A	Pot	Maize	Castel	CalciumNitrate_B	Synthetic N	noP	Control	4
UNINAb_2013a	UNINAb_2013a_B	Pot	Maize	Castel	CalciumNitrate_B	Synthetic N	TSP_A	Soluble P	4
UNINAb_2013a	UNINAb_2013a_C	Pot	Maize	Castel	CalciumNitrate_B	Synthetic N	RP_A	Soluble P	4
UNINAb_2013a	UNINAb_2013a_D	Pot	Maize	Castel	CompMan_D	Organic N	CompMan_D-RP_A	Rock P	4
UNINAb_2013a	UNINAb_2013a_E	Pot	Maize	Castel	CompMan_E	Organic N	CompMan_E	Manure	4
UNINAb_2014a	UNINAb_2014a_A	Pot	Maize	Castel	CalciumNitrate_B	Synthetic N	noP	Control	4
UNINAb_2014a	UNINAb_2014a_B	Pot	Maize	Castel	CompMan_F	Organic N	CompMan_F	Manure	5
UNINAb_2014b	UNINAb_2014b_A	Field	Maize	Castel	HAST	Synthetic N	noP	Control	1
UNINAb_2014b	UNINAb_2014b_B	Field	Maize	Castel	HAST	Synthetic N	TSP_D	Soluble P	1
UNINAb_2014b	UNINAb_2014b_C	Field	Maize	Castel	CompMan_H	Organic N	CompMan_H	Manure	1
UNINAb_2015a	UNINAb_2015a_A	Field	Maize	Castel	HAST	Synthetic N	noP	Control	1
UNINAb_2015a	UNINAb_2015a_B	Field	Maize	Castel	HAST	Synthetic N	TSP_D	Soluble P	1
UNINAb_2015a	UNINAb_2015a_C	Field	Maize	Castel	CompMan_G	Organic N	CompMan_G	Manure	1
UNINAb_2015b	UNINAb_2015b_A	Pot	Maize	Castel	CalciumNitrate_B	Synthetic N	noP	Control	2
UNINAb_2015b	UNINAb_2015b_B	Pot	Maize	Castel	CalciumNitrate_B	Synthetic N	TSP_C	Soluble P	2
UNINAb_2015b	UNINAb_2015b_C	Pot	Maize	Castel	CalciumNitrate_B	Synthetic N	RP_F	Rock P	2
UNINAb_2016a	UNINAb_2016a_A	Field	Maize	Castel	AmmoniumSulphate	Ammonium	noP	Control	4
UNINAb_2016a	UNINAb_2016a_B	Field	Maize	Castel	AmmoniumSulphate	Ammonium	RP_E	Rock P	4
