# Supplementary material for: Effectiveness of bio-effectors on maize, wheat and tomato performance and phosphorus acquisition from greenhouse to field scales in Europe and Israel: a meta-analysis
Source: Front Plant Sci. 2024 Apr 2;15:1333249. doi: 10.3389/fpls.2024.1333249 (PMC11020074; doi:10.3389/fpls.2024.1333249)
Supplement: Supplementary Table 3 — List of crops included + cultivars + information on suppliers [file DataSheet_3.pdf]

Nr.	Name	BE Type	Content(s)	Source
1	A. chroococcum 76A	Single bacterium	*Azotobacter chroococcum* strain 76A	UNAP b, University of Naples, Group a, Italy
2	AzoF	Single bacterium		Corvinus University of Budapest, Hungary?
3	B. simplex R41 ("ABI02S")	Single bacterium	*Bacillus simplex* strain R41	ABiTEP GmbH, Berlin, Germany
4	Bacillus amyloliquefaciens ABI01	Single bacterium	*B. amyloliquefaciens* strain ABI01	ABiTEP GmbH, Berlin, Germany
5	Bacillus atrophaeus ABI02A	Single bacterium	B. atrophaeus, strain ABI02A	ABiTEP GmbH, Berlin, Germany
6	Bacillus atrophaeus ABI05	Single bacterium	*B. atrophaeus* , strain ABI05	ABiTEP GmbH, Berlin, Germany
7	Easy Start TE-Max	Single bacterium	*Bacillus subtilis* E4-CDX An ingredient in the product Easy Start + E4-CDX)	COMPO EXPERT GmbH, Münster, Germany
8	FZB45 (ABI21)	Single bacterium	*Bacillus amyloliquefaciens* strain FZB45	ABiTEP GmbH, Berlin, Germany
9	Herbaspirillum sp.	Single bacterium	Strain of *Herbaspirillum sp.*	University of Hohenheim, Group a, Germany
10	P. mucilaginosus ("Muci")	Single bacterium	Strain of *Paenibacillus mucilaginosus*	ABiTEP GmbH, Berlin, Germany
11	Pf153	Single bacterium	*Pseudomonas sp.,* strain PF153	FiBL, Research Institute of Organic Agriculture, Frick, Switzerland
12	Proradix	Single bacterium	*Pseudomonas sp.* strain DSMZ 13134	Sourcon Padena GmbH, Tübingen, Germany
13	Rhizovital 42	Single bacterium	Bacillus amyloliquefaciens strain FZB42	ABiTEP GmbH, Berlin, Germany
14	RU47	Single bacterium	*Pseudomonas jessenii* strain RU47	JKI, Julius Kühn-Institut, Braunschweig, Germany
15	Biol. fertilizer DC	Single fungus	*Penicillium sp* ., strain PK 112	Prophyta GmbH now owned by Byer CropScience Biologics GmbH, Monheim am Rhein, Germany
16	Biol. fertilizer OD	Single fungus	*Penicillium* sp., strain PK 112	Prophyta GmbH now owned by Byer CropScience Biologics GmbH, Monheim am Rhein, Germany
17	G. interradices	Single fungus	Strain of *Glomus interadices*	Anhalt University of Life Sciences, Köthen, Germany
18	L. pygmaeum	Single fungus	Strain of *Lachnum pygmaeum*	Anhalt University of Life Sciences, Köthen, Germany
19	L. virgineum	Single fungus	Strain of *Lachnum virgineum*	Anhalt University of Life Sciences, Köthen, Germany
20	MTD	Single fungus	Strain of *Trichoderma sp.*	Corvinus University of Budapest, Hungary
21	OMG08 + ZnMn	Single fungus	*T. harzianum* strain OMG08 + Mn/Zn	Anhalt University of Life Sciences, Köthen, Germany
22	P. indica	Single fungus	Strain of *Piriformospora indica*	Anhalt University of Life Sciences, Köthen, Germany
23	T. harzianum OMG08	Single fungus	*Trichoderma harzianum* strain OMG08	Anhalt University of Life Sciences, Köthen, Germany/Prophyta?
24	T. harzianum OMG16	Single fungus	*Trichoderma harzianum* strain OMG16	Anhalt University of Life Sciences, Köthen, Germany
25	T. virens M9B	Single fungus	*Trichoderma virens* isolated from maize roots	Anhalt University of Life Sciences, Köthen, Germany
26	T. virens RapsB5	Single fungus	*Trichoderma virens* strain RapsB5, isolated from rape	Anhalt University of Life Sciences, Köthen, Germany
27	Trianum-P	Single fungus	*Trichoderma harzianum strain T22*	Koppert B.V., Berkel en Rodenrijs, Netherlands
28	Trichoderma-WG	Single fungus	Strain of *Trichoderma harzianum*	Prophyta GmbH now owned by Byer CropScience Biologics GmbH, Monheim am Rhein, Germany
29	TrichoStar	Single fungus	*Trichoderma harzianum* strain T58	Gerlach Ltd now part of Intrachem Bio Deutschland GmbH & Co. KG, Bad Camberg, Germany
30	Vitalin AM	Single fungus	Strain of *Glomus interadices*	Vitalin Pflanzengesundheit GmbH, Ober-Ramstadt, Germany
31	Vitalin T50	Single fungus	Strain of *Trichoderma harzianum*	Vitalin Pflanzengesundheit GmbH, Ober-Ramstadt, Germany
32	Aegis ("MYC")	Mixture	*Rhizophagus irregularis, Funneliformis mossae*	Italpollina, Aschau im Chiemgau , Germany
33	Agrinos HytA	Mixture	*Azotobacter sp* fermentation product 22 different bacreria and fungi + seaweed extract + plant extract	Agrinos, USA
34	Agrinos HytB	Mixture	Fermentation product amino acids peptides micro nutrients	Agrinos, USA

35	B. a.+ seaw. extract, seed coating	Mixture	*B. amyloliquefaciens* /seaweed extract (seed coating)	Limagrain GmbH, Edemissen, Germany
36	B. simplex R41+AlgaVyt Zn/Mn	Mixture	*Bacillus simplex* strain R41 + AlgaVyt Zn/Mn	ABiTEP GmbH, Berlin, Germany + Agriges SRL, Ponte, Italy
37	Bacillus spec.	Mixture	*Bacillus spp.* Strains	ABiTEP GmbH, Berlin, Germany
38	BactoConc	Mixture	5 Bacillus strains	Anhalt University of Life Sciences, Köthen, Germany
39	BactoFil	Mixture	Strains of Azospirillum brasilense, Azotobactoer vinerolandii, Bacillus megaterium, P. fluorescens, Streptomyces albus	Corvinus University of Budapest, Hungary
40	BactoProf	Mixture	5 *Bacillus* strains/*Trichoderma sp* . strain/humic extracts	Anhalt University of Life Sciences, Köthen, Germany
41	Biorex 1	Mixture	Strains of *Bacillus subtilis, B. thuringiensis, B. megaterium*	Italpollina, Aschau im Chiemgau , Germany
42	Biorex 1 + T. harzianum OMG16	Mixture	Biorex 1 + *T. harzianum* strain OMG16	Italpollina, Aschau im Chiemgau , Germany + Anhalt University of Life Sciences, Köthen, Germany
43	Biorex 1 + T. virens RapsB5	Mixture	Biorex 1 + *T. virens* strain RapsB5	Italpollina, Aschau im Chiemgau , Germany + Anhalt University of Life Sciences, Köthen, Germany
44	Biorex 2	Mixture	Strains of *Azotobacter chrococcum, Azospirillum lipoferum, Pseudomonas putida*	Italpollina, Aschau im Chiemgau , Germany
45	Biorex 2 + T. harzianum OMG16	Mixture	Biorex 2 + *T. harzianum* strain OMG16	Italpollina, Aschau im Chiemgau , Germany + Anhalt University of Life Sciences, Köthen, Germany
46	Biorex 2 + T. virens RapsB5	Mixture	Biorex 2 + *T. virens* strain RapsB5	Italpollina, Aschau im Chiemgau , Germany + Anhalt University of Life Sciences, Köthen, Germany
47	Biorex1 + Biorex2	Mixture	Biorex 1 + Biorex 2	Italpollina, Aschau im Chiemgau , Germany
48	Combifector A	Mixture	*T. harzianum* , strain OMG16 + 5 *Bacillus* strains + Mn/Zn	Anhalt University of Life Sciences, Köthen, Germany
49	Combifector A + HA from comp. artichoke	Mixture	Combifector A + HA extracted from compost made with artichoke	Anhalt University of Life Sciences, Köthen, Germany + UNINA
50	Combifector A + Nematec	Mixture	Combifector A + Nematec	Anhalt University of Life Sciences, Köthen, Germany + BioAtlantis Ltd., Co. Kerry, Ireland
51	Combifector B	Mixture	*T. harzianum* , strain OMG16 + 5 *Bacillus* strains + Mn/Zn	Anhalt University of Life Sciences, Köthen, Germany
52	Fermented nettle leaf solution	Mixture	Fermented nettle leaf solution	Corvinus University of Budapest, Hungary
53	FZB45+"Muci"	Mixture	*Bacillus amyloliquefaciens* strain FZB45 + Strain of *Paenibacillus mucilaginosus*	ABiTEP GmbH, Berlin, Germany
54	FZB45+TriWG	Mixture	*Bacillus amyloliquefaciens* strain FZB45 + strain of *Trichoderma harzianum*	ABiTEP GmbH, Berlin, Germany+Prophyta GmbH now owned by Byer CropScience Biologics GmbH, Monheim am Rhein, Germany
55	Geoagit	Mixture	Strains of *Pseudomonas, Streptomyces, Cellvibrio, Bacillus, Azotobacter, Azospirillum*	Geosan Környezetvédelmi Kft., Budapest, Hungary
56	MegaNit	Mixture	Strains of *Azotobacter chrococcum, Azospirillum spp., Bacillus megaterium, Bacillus subtilis*	Corvinus University of Budapest, Hungary
57	OMG08 + BactoConc	Mixture	*T. harzianum* strain OMG08 + BactoConc	Anhalt University of Life Sciences, Köthen, Germany
58	OMG08 + BactoConc + ZnMn	Mixture	*T. harzianum* strain OMG08 + BactoConc + Mn + Zn	Anhalt University of Life Sciences, Köthen, Germany
59	OMG16 + BactoConc	Mixture	*Trichoderma harzianum* strain OMG16 + BactoConc	Anhalt University of Life Sciences, Köthen, Germany

60	OMG16 + MnZn	Mixture	*Trichoderma harzianum* strain OMG16 + Mn + Zn	Anhalt University of Life Sciences, Köthen, Germany
61	OMG16 + Rhizovital 42 + AlgaVyt	Mixture	*Trichoderma harzianum* strain OMG16 + *Bacillus amyloliquefacien* s strain FZB42 + AlgaVyt	Anhalt University of Life Sciences, Köthen, Germany+ABiTEP GmbH, Berlin, Germany+Agriges SRL, Ponte, Italy
62	OMG16+BactoConc+ZnMn	Mixture	*Trichoderma harzianum* strain OMG16 + BactoConc + Mn + Zn	Anhalt University of Life Sciences, Köthen, Germany
63	P. williamsii isolate	Mixture	Two strains of *Piriformospora williamsi*	Anhalt University of Life Sciences, Köthen, Germany
64	Phylazonit	Mixture	Strains of *Azotobacter chrococcum, Bacillus megaterium*	Corvinus University of Budapest, Hungary
65	PromotPlus	Mixture	Strains of *T. harzianum, T. virens*	Gerlach Ltd now part of Intrachem Bio Deutschland GmbH & Co. KG, Bad Camberg, Germany
66	Proradix + Algafect	Mixture	*Pseudomonas sp.* strain DSMZ 13134 + AlgaFect	Sourcon Padena GmbH, Tübingen, Germany + Agriges SRL, Ponte, Italy
67	Proradix + AlgaVyt	Mixture	*Pseudomonas sp.* strain DSMZ 13134 + AlgaVyt	Sourcon Padena GmbH, Tübingen, Germany + Agriges SRL, Ponte, Italy
68	Proradix + AlgaVyt Zn/Mn	Mixture	*Pseudomonas sp* . strain DSMZ 13134 + AlgaVyt Zn/Mn	Sourcon Padena GmbH, Tübingen, Germany + Agriges SRL, Ponte, Italy
69	Proradix + Ecolicitor	Mixture	*Pseudomonas sp.* strain DSMZ 13134 + Ecolicitor	Sourcon Padena GmbH, Tübingen, Germany + BioAtlantis Ltd., Co. Kerry, Ireland
70	Proradix + HA from comp. artichoke	Mixture	*Pseudomonas sp* . strain DSMZ 13134 + HA extracted from compost made with artichoke	Sourcon Padena GmbH, Tübingen, Germany +UNINA
71	Proradix + Nematec	Mixture	*Pseudomonas sp* . strain DSMZ 13134 + Nematec	Sourcon Padena GmbH, Tübingen, Germany + BioAtlantis Ltd., Co. Kerry, Ireland
72	Proradix + SuperFifty	Mixture	*Pseudomonas sp* . strain DSMZ 13134 + SuperFifty	Sourcon Padena GmbH, Tübingen, Germany + BioAtlantis Ltd., Co. Kerry, Ireland
73	Proradix+ABI02A+Trichoderma-WG	Mixture	*Pseudomonas sp* . strain DSMZ 13134 + *B. atrophaeus* , strain ABI02A + strain of Trichoderma harzianum	Sourcon Padena GmbH, Tübingen, Germany + ABiTEP GmbH, Berlin, Germany + Prophyta GmbH now owned by Byer CropScience Biologics GmbH, Monheim am Rhein, Germany
74	Proradix+FZB45	Mixture	*Pseudomonas sp.* strain DSMZ 13134 + *Bacillus amyloliquefaciens* strain FZB45	Sourcon Padena GmbH, Tübingen, Germany + ABiTEP GmbH, Berlin, Germany
75	Proradix+FZB45+Trichoderma-WG	Mixture	*Pseudomonas sp.* strain DSMZ 13134 + *Bacillus amyloliquefaciens* strain FZB45 + strain of Trichoderma harzianum	Sourcon Padena GmbH, Tübingen, Germany + ABiTEP GmbH, Berlin, Germany + Prophyta GmbH now owned by Byer CropScience Biologics GmbH, Monheim am Rhein, Germany
76	Proradix+Rhizovital	Mixture	*Pseudomonas sp* . strain DSMZ 13134 + *Bacillus amyloliquefaciens* strain FZB42	Sourcon Padena GmbH, Tübingen, Germany + ABiTEP GmbH, Berlin, Germany
77	Proradix+VitalinAM	Mixture	*Pseudomonas s* p. strain DSMZ 13134 + VitalinAM	Sourcon Padena GmbH, Tübingen, Germany + Vitalin Pflanzengesundheit GmbH, Ober-Ramstadt, Germany
78	RhizoVital + Eco-T	Mixture	*B. amyloliquefaciens* strain FZB42 + Strain of *T. harzianum*	ABiTEP GmbH, Berlin, Germany
79	Rhizovital 42 + Biorex 2	Mixture	*B. amyloliquefaciens* strain FZB42 + Biorex 2	ABiTEP GmbH, Berlin, Germany + Italpollina, Aschau im Chiemgau , Germany
80	Rhizovital 42 + P. mucilaginosus ("Muci")	Mixture	*B. amyloliquefaciens* strain FZB42 + strain of *P. mucilaginosus*	ABiTEP GmbH, Berlin, Germany
81	Rhizovital 42 + RU47	Mixture	*B. amyloliquefaciens* strain FZB42 + *P. jessenii* strain RU47	ABiTEP GmbH, Berlin, Germany+JKI, Julius Kühn-Institut, Braunschweig, Germany
82	RhizoVital 42 + T. asperellum	Mixture	*B. amyloliquefaciens* strain FZB42 + *T. asperellum* strain kd	ABiTEP GmbH, Berlin, Germany

83	Rhizovital 42+Bacillus simplex R41	Mixture	*B. amyloliquefaciens* strain FZB42 + *Bacillus simplex* strain R41	ABiTEP GmbH, Berlin, Germany
84	Rhizovitial 42+HA from comp. manure	Mixture	*B. amyloliquefaciens* strain FZB42 + HA extracted from composted manure	ABiTEP GmbH, Berlin, Germany+UNINA
85	T. virens RapsB5 + BactoConc	Mixture	*T. virens* strain RapsB5 + BactoConc	Anhalt University of Life Sciences, Köthen, Germany
86	T. virens RapsB5 + BactoConc + MnZn	Mixture	*T. virens* strain RapsB5 + BactoConc + Mn/Zn	Anhalt University of Life Sciences, Köthen, Germany
87	T. virens RapsB5 + MnZn	Mixture	*T. virens* strain RapsB5 + Mn + Zn	Anhalt University of Life Sciences, Köthen, Germany
88	Trianum-P + Proradix + Rhizovital	Mixture	*Trichoderma harzianum* strain T22 + *Pseudomonas sp.* strain DSMZ 13134 + *Bacillus amyloliquefaciens* strain FZB42	Koppert B.V., Berkel en Rodenrijs, Netherlands + Sourcon Padena GmbH, Tübingen, Germany + ABiTEP GmbH, Berlin, Germany
89	Vitalin SP11	Mixture	Strains of *Bacillus subtilis, Pseudomonas sp., Streptomyces spp., HA, Extract of Ascophyllum nodosum*	Vitalin Pflanzengesundheit GmbH, Ober-Ramstadt, Germany
90	Algae95	Non-microbial	Extract of *Ascophyllum nodosum*	BioAtlantis Ltd., Co. Kerry, Ireland
91	AlgaFect	Non-microbial	Mixture of extracts from *Ascophyllum nodosum, Fucus spp. Laminaria spp., Spirulina sp.,* Nitrogen	Agriges SRL, Ponte, Italy
92	AlgaVyt	Non-microbial	Mixture of extracts from *Ascophyllum nodosum, Fucus spp. Laminaria spp., Spirulina sp.* , Nitrogen	Agriges SRL, Ponte, Italy
93	AlgaVyt Zn/Mn	Non-microbial	AlgaVyt + Zn/Mn	Agriges SRL, Ponte, Italy
94	BioA_A039F249	Non-microbial	Seaweed extract	BioAtlantis Ltd., Co. Kerry, Ireland
95	BioA_D002G138	Non-microbial	Seaweed extract	BioAtlantis Ltd., Co. Kerry, Ireland
96	Ecolicitor	Non-microbial	Extract of *Ascophyllum nodosum*	BioAtlantis Ltd., Co. Kerry, Ireland
97	HA from comp. municipal organic waste	Non-microbial	HA extracted from compost made with municipal organic waste	UNAP b, University of Naples, Group b, Italy
98	HA from composted artichoke	Non-microbial	HA extracted from compost made with artichoke	UNAP b, University of Naples, Group b, Italy
99	HA from composted manure	Non-microbial	HA extracted from compost made with manure	UNAP b, University of Naples, Group b, Italy
100	LamVita	Non-microbial	Seaweed extract	BioAtlantis Ltd., Co. Kerry, Ireland
101	Nematec	Non-microbial	Extract from *Laminaria spp.*	BioAtlantis Ltd., Co. Kerry, Ireland
102	P. indica extract	Non-microbial	Extract of *Pseudomonas indica*	Anhalt University of Life Sciences, Köthen, Germany
103	Prebiotic seaweed extract	Non-microbial	Seaweed extract	BioAtlantis Ltd., Co. Kerry, Ireland
104	Rygex	Non-microbial	Humic acids, seaweed extract, amino acids [8]	Agriges SRL, Ponte, Italy
105	Sorghum root extract	Non-microbial	Extract of sorghum roots	Anhalt University of Life Sciences, Köthen, Germany
106	SuperFifty	Non-microbial	Extract of *Ascophyllum nodosum*	BioAtlantis Ltd., Co. Kerry, Ireland
107	T. harzianum OMG08 extract	Non-microbial	Extract of *Trichoderma harzianum* strain OMG08	Anhalt University of Life Sciences, Köthen, Germany
