# Supplementary material for: Effectiveness of bio-effectors on maize, wheat and tomato performance and phosphorus acquisition from greenhouse to field scales in Europe and Israel: a meta-analysis
Source: Front Plant Sci. 2024 Apr 2;15:1333249. doi: 10.3389/fpls.2024.1333249 (PMC11020074; doi:10.3389/fpls.2024.1333249)
Supplement: Supplementary Table 4 — List of P and N fertilizers applied [file DataSheet_4.pdf]

Nr.	Crop	Cultivar	Source
1	Maize	Aapotheoz	-
2	Maize	Colisée	KWS SAAT SE & Co. Kga, Einbeck, Germany
3	Maize	Maxxis	RAGT Saaten Deutschland GmbH, Hiddenhausen, Germany
4	Maize	KXB 4132	-
5	Maize	Jessy	-
6	Maize	Kartagos	KWS SAAT SE & Co. Kga, Einbeck, Germany
7	Maize	LG 30.600	Limagrain Italia S.p.A, Fidenza, Italy
8	Maize	Andreea	Lovrin, Italy
9	Maize	Ronaldinio	KWS SAAT SE & Co. Kga, Einbeck, Germany
10	Wheat	Alex	-
11	Wheat	Aranka	-
12	Wheat	Scirocco	KWS SAAT SE & Co. Kga, Einbeck, Germany
13	Wheat	Ciprian	Lovrin, Italy
14	Wheat	Creso	CIMMYT, Italy
15	Wheat	Granary	Quantilagriculture Ltd., Lancashire, UK
16	Wheat	JB Diego	Senova, Cambridge, UK
17	Wheat	Kometus	Saatzucht Schweiger GbR, Moosburg, Germany
18	Tomato	Mobil	Kecskemet, Hungary
19	Tomato	Hellfrucht	Carl Sperling & Co. Saatzucht GmbH & Co. KG., Lüneburg, Germany
20	Tomato	Primadonna	Hazera Seeds, Israel
21	Tomato	Money Maker	-
22	Tomato	Microtrom	-
23	Tomato	Vulcan	-
24	Tomato	Brigit	-
