# Supplementary material for: Effectiveness of bio-effectors on maize, wheat and tomato performance and phosphorus acquisition from greenhouse to field scales in Europe and Israel: a meta-analysis
Source: Front Plant Sci. 2024 Apr 2;15:1333249. doi: 10.3389/fpls.2024.1333249 (PMC11020074; doi:10.3389/fpls.2024.1333249)
Supplement: Supplementary Table 5 — List of soils used with basic info on pH, P content, texture, %C. [file DataSheet_5.pdf]

Nr.	Fertilizer ID for meta-analysis	Year	Partner	Producer	Description
1	TSP_A	-	FiBL [12]	Landor, Switzerland	P fertilizer
2	TSP_B	2014	CULS [03]	Agropodnik Hradec Králové	Granules ca. 5 mm
3	TSP_C	-	HKKALKE [15]	AmFert	Triple Super Phosphate (Calcium dihydrogen phosphate)
4	TSP_D	2015	UNINAb [07b]	-	TSP
5	TSP_E	2015	ARO [20]	ICL	Triple-superphosphate
6	SP_A	2013	ARO [20]	ICL	superphosphate
7	CalciumdiHP		Choose from list	-	Lab grade Ca(H2PO4)2);
8	Phosphate	2014	AFBI [09]	-	-
9	MAP	2014	UHOHb [01b]	Yara GmbH, Germany	Krista™ MAP; Monoammonium phosphate (12 % NH4-N, 22 % P)
10	DAP	2014	UHOHb [01b]	Compo?	18 % N, 46 % P2O5 ≙ 20 % P
11	Duratec	-	UHOHa [01a]	Compo GmbH & Co. KG	Granular solid fertilizer, surface-treated, 30% polymercoated
12	EasyStart	2014	UHOHb [01b]	Compo Expert, Münster, Germany	Mono-ammonium phosphate (11 % NH4-N, 21 % P) plus 0.6 % Fe, 0.1% Mn, 1.0 % Zn,
13	EasyStartE4	2014	UHOHb [01b]	Compo Expert, Münster, Germany	Bacillus subtilis (0.15%) +Mono-ammonium phosphate (11 % NH4-N, 21 % P) plus 0.6 % Fe, 0.1% Mn, 1.0 % Zn,
14	NPK_A		BUAS	Azomures Targu-Mures	-
15	NP	-	ABiTEP	-	14% N, 37%P
16	RP_A	2013	FiBL [12]	Landor, Switzerland	"Rock phosphate", superphosphate
17	RP_B	2014	HKKALKE [15]	Herbert Molitor	Rock phosphate from Syria
18	RP_C	2014	Choose from list	SeNaPro GmbH	Rock phosphate from Syria
19	RP_D	-	UHOHb [01b]	-	Rock phosphate
20	RP_E	2016	FiBL [12]	Landor, Switzerland	Granules ca. 7 mm, Granuphos
21	RP_F	2015	UNINAb[07b]	-	Supplied by the local research station, very low solubility in water
22	BioAsh_A	2014	CULS [03]	-	wood ash
23	BioAsh_B	2014	CULS [03]	-	straw ash
24	SSA_A	2013	UCPH	Avedøre Rensningsanlæg	Sewage sludge ash
25	SSA_B	2009	FiBL [12]	ETHZ	Sewage sludge ash (MgSS-Ash)
26	SSA_C	2013	HKKALKE [15]	sewage incineration plant Bonn	sewage incineration ash from municipal sewage sludge of incineration plant Bonn
27	TP	2002	Choose from list	Luxengrais Luxembourg	Thomasphosphate (Basic Slag)
28	SSA-BOFS	2011	Choose from list	Salzgitter Flachstahl GmbH	molten BOF slag enriched with sewage sludge ash
29	BioChar_A	2015	CULS [03]	-	wood biochar
30	SS_A	2013	UCPH	Avedøre Rensningsanlæg, Denmark	Dewatered and degassed sewage sludge
31	SS_B	2015	CULS [03]	-	sewage sludge
32	SS_C	2013	HKKALKE [15]	sewage treatment plant Bonn	municipal sewage sludge from sewage treatment plant in Bonn
33	SS_D	2015	UHOHa [01a]	ALKALOIDA Chemicals Co. Ltd.	Industrial by-product; cooperation with an institute in Hungary; Brigitta Toth; (poppy shell-based alkaloid production)
34	GSS	2014	HKKALKE [15]	sewage incineration plant Bonn	Dried and granulated sewage sludge
35	PSS	2015	CULS [03]	-	pyrolysed sewage sludge
36	TSS	2015	CULS [03]	-	torrefied sewage sludge

37	Struvite	-	UHOHb [01b]	MAP Berlin	Struvite, recylced form waste water by crystaliization (19.5 % P) (obtained from Improve-P project, Hohenheim)
38	Dig_A	2015	FiBL [12]	Gärgut Pratteln	solid digestate from biogas plant
39	Dig_B	2015	CULS [03]	-	solid fraction of digestate - separate
40	Dig_C	2014	HKKALKE [15]	Reterra	Digestate, dried and pelletized ("Humerra Gärprodukt pelletiert") http://www.reterra.de/ret/spektrum/duenger/humerra/landwirtschaft/aktivkompost_2424000/
41	Man_A	2013	UCPH	Local farm	Decanter centrifuge fiber fraction of pig manure
42	Man_B	2014	FiBL [12]	Local farm	Sheep manure
43	Man_C	2014	FiBL [12]	Local farm	Pork and chicken slurry
44	Man_D	2013	UHOHb [01b]	Kleinhohenheim	Sheep manure (Kleinhohenheim)
45	Man_E	2014	BUAS	Pig manure from private company	Pig manure from private company
46	Man_F	2015	BUAS	Pig manure from private company	Pig manure from private company
47	Man_G	2016	BUAS	Pig manure from private company	Pig manure from private company
48	CompMan_A	2015	AGRIGES [21]	Agriges	composted chicken manure pellets
49	CompMan_A-DMPP	2014	AGRIGES [21]	Agriges	composted chicken manure pellets + DMPP (1% of total N)
50	CompMan_B	2015	FiBL [12]	FiBL farm	Composted farm yard manure
51	CompMan_C	2015	UHOHa [01a]	Kleinhohenheim	Composted cow manure, research station Kleinhohenheim (data to be checked with Nino, 2015h)
52	CompMan_C-DMPP	2015	UHOHa [01a]	Kleinhohenheim	Composted cow manure, research station Kleinhohenheim + DMPP (1% of total N)
53	CompMan_D	2013	UNINAb [07b]	Local farm	cow manure compost produced on farm
54	CompMan_E	2013	UNINAb [07b]	Local farm	horse manure compost produced on farm
55	CompMan_F	-	UNINAb [07b]	Local farm	cow manure compost produced on farm
56	CompMan_G	2015	UNINAb [07b]	Local farm	cow manure compost produced on farm
57	CompMan_H	2014	UNINAb [07b]	Local farm	cow manure compost produced on farm
58	CompMan_I	2013	ARO [20]	Local farm	compost of cattle manure
59	Comp_A	2013	UCPH [8]	KomTek	Mature compost consisting of 42 % garden park waste, 36 % sewage sludge, 14 % straw and horse manure, 8 % wood mass
60	Comp_B	2014	FiBL [12]	Leureco	Compost from green waste
61	Comp_C	2014	HKKALKE [15]	Reterra	Green cuttings compost from a municipal composting plant
62	Comp_D	-	UHOHa [01a]	ALKALOIDA Chemicals Co. Ltd.	Industrial by-product; cooperation with an institute in Hungary; Brigitta Toth
63	Hornmeal	-	FiBL [12]	Hauert	Grounded hourn
64	OrgFert_A	-	CUB [05]	GARMED Kft.	Viano, NPP=5-5-8 +3Mg organic fertilizer
65	OrgFert_B	2013	BUAS [04]	Local farm?	Manure
66	OrgFert_C	2015	BUAS [04]	Local farm?	Manure
67	OrgFert_D	2016	BUAS [04]	Local farm?	Manure
68	AN_A	2015	CULS [03]	Lachner	Ammonium Nitrate Granules ca. 1 mm, applied in solution
69	AN_B	-	UNINAa [07a]	-	Ammonium Nitrate
70	CalciumNitrate_A	2013	CULS [03]	Lovochemie Lovosice	Granules app. 5 mm

71	CalciumNitrate_B	-	UHOHb [01b]	Sigma Aldrich	Ca(NO3)2 (Laboratory grade), eg. SigmaAldrich
72	CalciumNitrate_C	-	HKKalke [15]	-	Calcium nitrate [Ca(NO3)2 x 4H2O]
73	CAN_A	2013	UCPH	Yara Liva	Calcinit
74	CAN_B	2014	CULS [03]	Lovochemie Lovosice	Granules ca. 5 mm - to all treatments
75	CAN_C	-	AFBI [09]	-	-
76	HAST	-	UHOHa [01a]	-	Urea=46%N
77	UAN	2015	CULS [03]	Agropodnik Hradec Králové	Liquid (Urea ammonium nitrate)
78	NovaTec	2014	UHOHb [01b]	Compo Expert, Münster, Germany	NovaTec Solub 21; Stabilized (NH4)2SO4 (21 % NH4-N, 24 % S)
79	MinA	2014	UHOHb [01b]	Compo Expert, Münster, Germany	Stabilized (NH4)2SO4 (21 % NH4-N, 24 % S); + MgSO4; K2SO4;
80	MinN	-	UHOHa [01a]	Compo?	Not a commercial product: Ca(NO3)2; + MgSO4; K2SO4;
81	NP_A	2015	ABI [14]	-	NP (14:37)
82	NP_B	2016	UNINA [07a]	-	NP (18:46)
83	ActivN	-	ABI	-	N-P-K: 16-5-8
84	Sulfammo32	-	ABI	Timac Agro	-
85	AmmoniumSulphate	-	HKKalke [15]	-	Ammonium sulfate [(NH4)2SO4]
86	ASN_CAN	2016	UHOHa [01a]	-	ammonium sulphate nitrate (ASN) + calcium ammonium (CAN)
87	Potash	2014	AFBI [09]	-	-
88	KCl	-	CULS [03]	-	-
89	Patentkali_A	2013	UCPH [08]	-	Potassium fertilizer
90	Patentkali_B	2013	FiBL [12]	K+S KALI GmbH	SKMg fertilizer
91	Patentkali_C	2013	CULS [03]	Agrofert	Crystals ca. 5 mm
92	Patentkali_D	-	CUB [05]	-	K2SO4+Mg 30 (+10+17); 3o%watersolubile K(=25%K); 10%MgO ws Mg (=6,1% Mg); 17% S ws.
93	Patentkali_E	-	CUB [05]	-	-
94	Kalisulfate_A	2014	FiBL [12]	Landor	SK-fertilizer
95	Kalisulfate_B	-	UHOHb [01b]	-	Lab grade K2SO4
96	Kalisulfate_C	2015	UNINAb [07b]	-	Used for field trial
97	kalimagnesia	-	UNINAb [07b]	-	-
98	Lithovit	2013	BUAS [04]	zeovita GmbH, Roter	contain: >75% CaCO3, >4% MgCO3, >0,25% Fe, >5,0% SiO2, >0,1% K2O, >0,015% N, >0,015% P2O5, >0,01% Mn; dose 2-2,5 kg/ha; 3 applications interval of 30 days
99	Multi-K	2013	BUAS [04]	Haifa Chemicals Ltd. Israel	contain N 13% and K2O 46%; dose 8-13 kg/ha respectively 0,2-0,3% w/w
100	Solution_A	-	UCPH [08]	-	Nutrient solution containing N, K and Ca
101	Solution_B	-	UNINAa [07a]	-	6:18:36 + me
102	Solution_C	-	UNINAa [07a]	-	6:18:36 + me +Amm. Nit. half strenght)
103	Solution_D	-	UNINAa [07a]	-	6:18:36 + me + Amm. Nit.
104	Solution_E	-	UNINAa [07a]	-	20:20:20 + me 100%
105	Solution_F	-	UNINAa [07a]	-	20:20:20 + me 70%
106	Solution_G	-	UNINAa [07a]	-	20:20:20 + me (half strenght)
107	Solution_H	-	UNINAa [07a]	-	20:20:20 + me + Ammonium nitrate 27%

108	Solution_I	-	ARO [20]	-	-
109	noP	-		-	No addition of P fertilizer
110	noN	-		-	No addition of N fertilizer
111	noK	-		-	No addition of K fertilizer
112	RP_D-Man_D	-	UHOHb [01b]	-	A mixture of rock P and manure
113	AS-Man_D	-	UHOHb [01b]	-	A mixture of ammonium sulphate and manure
114	CaN-Man_D	-	UHOHb [01b]	-	A mixture of calcium nitrate and manure
115	AN_A-SS_B	-	CULS [03]	-	A mixture of AN_A & SS_B
116	AN_A-BioChar_A	-	CULS [03]	-	A mixture of AN_A & BioChar_A
117	CAN_A-Comp_B	-	FiBL [12]	-	A mixture of CAN_A & Comp_B
118	CAN_A-CompMan_A	-	FiBL [12]	-	A mixture of CAN_A & CompMan_A
119	CAN_A-Dig_A	-	FiBL [12]	-	A mixture of CAN_A & Dig_A
120	CAN_A-CompMan_B	-	FiBL [12]	-	A mixture of CAN_A & CompMan_B
121	CAN_A-SS_A	-	UCPH [08]	-	A mixture of CAN_A & SS_A
122	CAN_A-Man_A	-	UCPH [08]	-	A mixture of CAN_A & Man_A
123	CAN_A-Comp_A	-	UCPH [08]	-	A mixture of CAN_A & Comp_A
124	Hornmeal-Comp_B	-	FiBL [12]	-	A mixture of Hornmeal & Comp_B
125	AN_A-PSS	-	CULS [03]	-	A mixture of AN_A & PSS
126	AN_A-SS_B-a	-	CULS [03]	-	A mixture of AN_A & SS_B
127	AN_A-SS_B-b	-	CULS [03]	-	A mixture of AN_A & SS_B
128	AN_A-TSS-a	-	CULS [03]	-	A mixture of AN_A & TSS
129	AN_A-TSS-b	-	CULS [03]	-	A mixture of AN_A & TSS
130	AN_A-Dig_B	-	CULS [03]	-	A mixture of AN_A & Dig_B
131	AN_A-BioAsh_A	-	CULS [03]	-	A mixture of AN_A & BioAsh_A
132	AN_A-BioAsh_B	-	CULS [03]	-	A mixture of AN_A & BioAsh_B
133	CAN_A-OrgFert_A	-	CUB [05]	-	-
134	HAST-NP_B	-	UNINAa [07a]	-	-
135	CalciumNitrate_C-SS_C	-	HKKALKE [15]	-	-
136	CalciumNitrate_C-Comp_C	-	HKKALKE [15]	-	-
137	AmmoniumSulphate-Dig_C	-	HKKALKE [15]	-	Have written to Martin Rex regarding the composition of the Humerra product
138	Sulfammo32-ActivN	-	ABI	-	-
139	HAST-NP_A	-	ABI	-	-
140	CalciumNitrate_C-GSS	-	HKKALKE [15]	-	-
141	NovaTec-DAP	-	UHOHAa [01a]	-	-
142	Man_E-NPK_A	-	BUAS [04]	-	A mixture of Man_E and NPK_A
143	Man_F-NPK_A	-	BUAS [04]	-	A mixture of Man_F and NPK_A
144	Man_G-NPK_A	-	BUAS [04]	-	A mixture of Man_F and NPK_A
145	CompMan_D-RP_A	-	UNINAb [07b]	-	A mixture of CompMan_D and RP_A
