# Supplementary material for: Effectiveness of bio-effectors on maize, wheat and tomato performance and phosphorus acquisition from greenhouse to field scales in Europe and Israel: a meta-analysis
Source: Front Plant Sci. 2024 Apr 2;15:1333249. doi: 10.3389/fpls.2024.1333249 (PMC11020074; doi:10.3389/fpls.2024.1333249)
Supplement: Supplementary file 6 [file DataSheet_6.pdf]

Nr.	Soil ID for the meta-analysis	Country	Partner	Management	Year	Laboratroy for analysis	pH determination	pH	CAL-P (mg P/kg)	Water-P (mg P/kg)	Olsen-P (mg P/kg)	Texture	Soil organic carbon (%)
1	1	Czech Republic	CULS [03]	-	2016	Local lab	0.01M CaCl2	7.77	-	-	30.10	-	-
2	2	Czech Republic	CULS [03]	-	2016	Local lab	0.01M CaCl2	7.62	-	-	66.60	-	-
3	14	Czech Republic	CULS [03]	-	2016	Local lab	0.01M CaCl2	7.85	-	-	28.30	-	-
4	101	Czech Republic	CULS [03]	-	2016	Local lab	0.01M CaCl2	7.89	-	-	43.20	-	-
5	Barvendorf_A	Germany	UHOHb [01b]	Low-P silt loam luvisol (Ap horizon of Luvisol, Barvendorf, Konstans, Germany)	2015	Local lab	0.01M CaCl2	6.40	7.00	-	8.00	Silt loam	2.8
6	Bavendorf_B	Germany	UHOHa [01a]	organic farming	?	LA Chemie (UHOH)	0.01M CaCl2	6.10	13.96	-	19.60	clay loam	1.87
7	Baven_B+Kleinh_D	Germany	UHOHa [01a]	mix of both soils	-	LA Chemie (UHOH)	0.01M CaCl2	6.87	37.64	-	38.94	Silty clay loam	1.74
8	Bayern	Germany	JKI [02]	-	2016	LUFA Speyer	-	7.58	-	-	-	-	-
9	Buus	Switzerland	FiBL [12]	organic	2013	LA Chemie (UHOH)	0.01M CaCl2	6.60	6.55	na	20.70	Silty Clay Loam	2.64
10	Castel	Italy	UNINAb [07b]	Vertic Xerofluvent	2013	LA Chemie (UHOH)	0.01M CaCl2	7.30	44.00	-	11.00	Clay loam	1.5
11	Chernozem	Germany	AUAS [11]	3 years fallow soil	2013	UHOH	0.01M CaCl2	7.40	150.00	na	21.90	Silt loam	2.36
12	C-Loess_A	Germany	UHOHa [01a]	subsoil loess with low organic matter and high carbonate/pH	-	LA Chemie (UHOH)	0.01M CaCl2	7.70	6.55	-	-	Loam	-
13	C-Loess_B	Germany	UHOHb [01b]	C-horizon loess subsoil	-	Local lab	0.01M CaCl2	7.60	-	-	1.00	Loam	0.3
14	CRUCIAL-CMA	Denmark	UCPH [08]	Continously cropped fertilization with cattle manure (high rate) since 2003	2015	Local lab	water	7.00	-	33.10	-	Sandy loam	2.38
15	CRUCIAL-U_A	Denmark	UCPH [08]	Continously cropped without fertilization since 2003	2013	LA Chemie (UHOH)	0.01M CaCl2	5.90	16.58	-	-	Sandy loam	-
16	CRUCIAL-U_B	Denmark	UCPH [08]	Continously cropped without fertilization since 2003	2015	Local lab	water	7.20	-	2.90	-	Sandy loam	1.46
17	CUB_A	Hungary	CUB [05]	esological conditions	2014	Local lab	water	7.35	187.65	-	-	Sandy loam	0.98
18	CUB_B	Hungary	CUB [05]	esological conditions	2015	Local lab	water	7.53	297.41	-	-	Sandy loam	0.98
19	CUB_C	Hungary	CUB [05]	esological conditions	2016	Local lab	water	7.75	294.76	-	-	Sandy loam	0.98
20	DOK-M	Switzerland	FiBL [12]		2013	LA Chemie (UHOH)	0.01M CaCl2	5.70	23.13	-	-	Silt Loam	1.3
21	Dompierre	Switzerland	FiBL [12]	clover-grass meadow	2015	Local lab	0.01M CaCl2	7.83	-	-	-	Sandy Loam	1.74
22	Hagenwil	Switzerland	FiBL [12]	clover-grass meadow	2014	Local lab	-	-	-	-	-	-	-
23	HeiHo	Germany	UHOHa [01a]	Soil from Filderlehm	2015	LA Chemie (UHOH)	0.01M CaCl2	7.10	100.40	-	-	Silty Clay Loam	1.38
24	High P-sorbing Soil	Germany	UHOHb [01b]	25% CaCO3+ 75% Long-term unfertilized low-P grassland soil	2013	LA Chemie (UHOH)	-	-	30.00	-	-	Loam	2.41
25	Horb_A	Germany	UHOHa [01a]	Soil from Horb	2015-2016	LA Chemie (UHOH)	0.01M CaCl2	7.10	65.46	-	-	Silty clay	2.42
26	Horb_B	Germany	UHOHa [01a]	Soil from Horb am Neckar (private farmer´s site)	2016	LA Chemie (UHOH)	0.01M CaCl2	6.50	52.00	-	-	Sandy clay loam	-
27	Horb_C	Germany	UHOHa [01a]	Filderlehm (Heildfeldhof, Uni Hohenheim)	1993	LA Chemie (UHOH)	0.01M CaCl2	6.97	82.21	-	-	Silt loam	1.35
28	Humpolec_A	Czech republic	CULS [03]	-	2013	LA Chemie (UHOH)	0.01M CaCl2	5.20	42.31	10.06	-	Loamy sand	1.63
29	Humpolec_B	Czech Republic	CULS [03]	-	2014	Local lab	0.01M CaCl2	4.50	59.00	9.00	-	Loam	1.45
30	Humpolec_C	Czech republic	CULS [03]	-	2013	LA Chemie (UHOH)	0.01M CaCl2	5.20	45.04	11.20	-	Loamy sand	1.63
31	Humpolec_D	Czech republic	CULS [03]	-	2015	LA Chemie (UHOH)	0.01M CaCl2	5.30	52.15	12.40	-	Loamy sand	1.63
32	Humpolec_E	Czech republic	CULS [03]	-	2016	LA Chemie (UHOH)	0.01M CaCl2	5.20	43.79	10.01	-	Loamy sand	1.63
33	IHOF_A	Germany	UHOHb [01b]	Field soil; sugar beet was the previous crop in rotation	2014	LA Chemie (UHOH)	0.01M CaCl2	6.90	119.00	-	78.70	Silt loam	1.1
34	IHOF_B	Germany	UHOHb [01b]	-	-	LA Chemie (UHOH)	0.01M CaCl2	7.00	82.90	-	55.80	Silt loam	1.07
35	IHOF_C	Germany	UHOHa [01a]	Soil from Ihinger Hof renningen	2014	LA Chemie (UHOH)	0.01M CaCl2	6.50	83.00	-	-	Silty Clay Loam	1.23
36	IHOF_D	Germany	UHOHa [01a]	Soil from Ihinger Hof renningen (IHOF 2016)	2016	LA Chemie (UHOH)	0.01M CaCl2	7.10	82.92	-	-	Silty Clay Loam	1.28
37	IHOF_E	Germany	UHOHa [01a]	Soil from Ihinger Hof - Renningen	2014	LA Chemie (UHOH)	0.01M CaCl2	6.90	122.00	-	-	Silt loam	-
38	IHOF_F	Germany	UHOHa [01a]	-	2016	LA Chemie (UHOH)	0.01M CaCl2	7.00	87.20	-	60.40	-	-
39	JR	Germany	UHOHa [01a]	substrate mix from 30% soil, 60% peat, 10% sand	2013	LA Chemie (UHOH)	0.01M CaCl2	-	-	-	-	-	-
40	JR + Manure	Germany	UHOHa [01a]	substrate mix from 45% manure, 30% soil, 15% peat, 10% sand	2013	LA Chemie (UHOH)	0.01M CaCl2	-	-	-	-	-	-
41	Karlsruhe_A	Germany	JKI [02]	-	2014	LA Chemie (UHOH)	0.01M CaCl2	7.45	-	-	32.90	Silt Loam	-
42	Karlsruhe_B	Germany	UHOHa [01a]	Soil from LTZ Augustenberg, Karlsruhe ("Karlsruhe 1")	2014	LA Chemie (UHOH)	0.01M CaCl2	7.50	37.10	-	-	Silt loam	-
43	Karlsruhe_B_sterilized	Germany	UHOHa [01a]	Soil from LTZ Augustenberg, Karlsruhe, after tyndallization	2014	LA Chemie (UHOH)	0.01M CaCl2	7.50	37.10	-	-	Silt loam	-
44	Karlsruhe_C	Germany	UHOHa [01a]	Soil from LTZ Augustenberg, Karlsruhe, equal to Karlsruhe 1 ("Karlsruhe 2")	2014	LA Chemie (UHOH)	0.01M CaCl2	7.50	37.10	-	-	Silt loam	-

45	KH + Manure	Germany	UHOHa [01a]	soil + 45 % sheep manure	2013	LA Chemie (UHOH)	0.01M CaCl2	-	-	-	-	-	-
46	Kleinhohenheim_A	Germany	UHOHb [01b]	("Kleinhohenheim")	2013	Local lab	0.01M CaCl2	6.90	35.00	-	-	Loam	-
47	Kleinhohenheim_B	Germany	UHOHb [01b]	Low-P soil from organic farming research station Kleinhohenheim ("Low-P Kleinhohenheim soil")	2014	LA Chemie (UHOH)	0.01M CaCl2	6.80	20.00	-	-	Loam	-
48	Kleinhohenheim_C	Germany	UHOHa [01a]	organic farming soil, agricultural research station Kleinhohenheim ("KH")	2013	LA Chemie (UHOH)	0.01M CaCl2	-	48.01	-	-	-	1.27
49	Kleinhohenheim_D	Germany	UHOHa [01a]	Research station Kleinhohenheim	2013	LA Chemie (UHOH)	0.01M CaCl2	6.10	69.82	-	49.80	Silt loam	1.3
50	Kleinhohenheim_E	Germany	UHOHa [01a]	Soil from research station Kleinhohenheim; 17.03.15 ("KH 92")	2015	LA Chemie (UHOH)	0.01M CaCl2	7.50	60.00	-	-	Silty clay	1.7
51	Kleinhohenheim_F	Germany	UHOHa [01a]	organic farming, arable land, Research station Kleinhohenheim ("KH_c")	2014	LA Chemie (UHOH)	0.01M CaCl2	7.00	36.70	-	-	Silty clay loam	-
52	Kleinhohenheim_G	Germany	UHOHb [01b]	Soil from organic farming research station Kleinhohenheim	2014	LA Chemie (UHOH)	0.01M CaCl2	6.90	57.00	-	-	Silty clay loam	1.7
53	Le-Caron	Switzerland	FiBL [12]	integrated	2013	LA Chemie (UHOH)	0.01M CaCl2	4.80	16.15	-	-	Silty Clay Loam	2.35
54	Lhota	Czech Republic	CULS [03]	-	2015	LA Chemie (UHOH)	0.01M CaCl2	5.50	160.00	-	-	Loam	2.36
55	Lipa	Czech republic	CULS [03]	-	2016	Local lab	0.01M CaCl2	5.90	46.00	5.00	40.00	Loamy sand	0.99
56	Low P-sorbing Soil	Germany	UHOHb [01b]	Long-term unfertilized low-P grassland soil	2013	LA Chemie (UHOH)	0.01M CaCl2	7.10	30.00	-	-	Loam	2.41
57	UHOH vineyard	Germany	UHOHa [01a]	low input grassland soil next to a vineyard	2013	LA Chemie (UHOH)	0.01M CaCl2	7.10	2.97	-	-	Silt loam	2.41
58	Lukavec_A	Czech republic	CULS [03]	-	2014	Local lab	0.01M CaCl2	5.40	48.00	11.20	35.10	Sandy loam	-
59	Lukavec_B	Czech republic	CULS [03]	-	2014	Local lab	0.01M CaCl2	5.40	47.12	12.15	-	Sandy loam	-
60	NDT-A	Denmark	UCPH [8]	Continous cropping, no P-fertilizer for app. 50 year	2013	LA Chemie (UHOH)	0.01M CaCl2	5.80	15.27	-	-	Sandy loam	1.01
61	NDT-E	Denmark	UCPH [8]	Continous cropping, standard N, P and K fert for app. 50 year	2013	LA Chemie (UHOH)	0.01M CaCl2	5.30	13.96	-	-	Loam	0.95
62	Newforge	Northern Ireland	AFBI [09]	Standard input field	2014	Local lab	water	5.80	-	-	-	Sandy loam	-
63	peat moss_UNINAa	Italy	UNINAa [07a]	-	2013	na	na	-	-	-	-	-	-
64	Podebrady	Czech Republic	CULS [03]	-	2014	Local lab	0.01M CaCl2	6.00	30.00	12.00	-	Sandy loam	1.76
65	Poyntzpass	Northern Ireland	AFBI [09]	Low input field	2014	Local lab	water	5.70	-	-	-	Loam	-
66	Ramat	Israel	ARO [20]	Ramat Hanegev	2013	LA Chemie (UHOH)	0.01M CaCl2	7.90	-	-	5.50	Sand	0.08
67	Romania_A	Romania	BUAS [04]	Greenhouse exp. 2013 Vertisol gleyed, decarbonated weak, on fluvial deposits, medium fine clay loam.	2013	LA Chemie (UHOH)	0.01M CaCl2	6.60	1200.00	-	2530.00	Clay loam	3.49
68	Romania_B	Romania	BUAS [4]	BUAS Field 2013 Maize A	2013	LA Chemie (UHOH)	0.01M CaCl3	5.80	87.00	-	-	-	1.75
69	Romania_C	Romania	BUAS [4]	BUAS Field 2013 Maize B	2013	LA Chemie (UHOH)	0.01M CaCl4	5.60	87.00	-	-	-	1.55
70	Romania_D	Romania	BUAS [4]	Green house exp. 2014 Vertisol gleyed, decarbonated weak, on fluvial deposits, medium fine clay loam.	2014	-	-	-	-	-	-	-	-
71	Romania_E	Romania	BUAS [4]	BUAS Field 2014 Wheat A	2014	-	-	-	-	-	-	-	-
72	Romania_F	Romania	BUAS [4]	BUAS Field 2014 Wheat B, Chermozem	2014	-	-	-	-	-	-	-	-
73	Romania_G	-	-	Green house experiment 2015	-	-	-	-	-	-	-	-	-
74	Romania_H	Romania	BUAS [4]	BUAS Field 2015 Wheat A, Chermozem	2015	-	-	-	-	-	-	-	-
75	Romania_I	Romania	BUAS [4]	BUAS Field 2015 Maize A, Chermozem	2015	-	-	-	-	-	-	-	-
76	Romania_J	-	-	Green house experiment 2016	-	-	-	-	-	-	-	-	-
77	Romania_K	Romania	BUAS [4]	BUAS Field 2016 Wheat A, Chermozem	2016	-	-	-	-	-	-	-	-
78	Romania_L	Romania	BUAS [4]	BUAS Field 2016 Maize A, Chermozem	2016	-	-	-	-	-	-	-	-
79	Romania_mix_A	Romania	BUAS [04]	Growing substrate for pre-culturing	2013	LA Chemie (UHOH)	0.01M CaCl2	6.70	3530.00	-	602.00	45% manure, 30%	8.27
80	Romania_mix_B	Romania	BUAS [04]	Growing substrate for pre-culturing	2014	-	-	-	3530.00	-	-	-	-
81	Romania_mix_C	Romania	BUAS [04]	Growing substrate for pre-culturing	2015	-	-	-	3530.00	-	-	-	-
82	Romania_mix_D	Romania	BUAS [04]	Substrate variant a1 - 45% manure, 30% garden soil, 15% peat, 10% sand	2016	-	-	-	3530.00	-	-	-	-
83	Romania_mix_E	Romania	BUAS [04]	Substrate variant a2 - 65% peat, 20% garden soil, 15% sand + DuraTec® Starter	2016	-	-	-	3530.00	-	-	-	-
84	Sanitz_A	Germany	JKI [02]	-	2016	LUFA Speyer	-	5.95	-	-	-	-	-
85	Sanitz_B	Germany	ABI [14]	-	2016	local lab	0.01M CaCl2	6.15	72.01	-	-	Sandy loam	1.13

86	TKS for pre-cultivation	Germany	UHOHa [01a]	pre-cultivation in peat substrate "TKS1 Instant Plus Aussaat und Pikieren" (Floragard Vertriebs GmbH für Gartenbau, Oldenburg, Deutschland) all values for nutrient contents in mg/L	NA	Local lab	Choose from list	-	34.91	-	-	-	-
87	UHOH campus	Germany	UHOHa [01a]	unfertilized grassland, UHOH campus	2013	LA Chemie (UHOH)	0.01M CaCl2	7.00	26.62	-	24.10	Silt Loam	1.99
88	UNINAa soil_A	Italy	UNINAa [07a]	-	2015	Local lab	water	?	-	-	-	Clay loam	-
89	UNINAa soil_B	Italy	UNINAa [07a]	With residual salt from the 2015 experiment	2016	Choose from list	water	6.50	-		-	Clay loam	-
90	Voerden	Germany	HKKALKE [15]	Arable land	2013	LA Chemie (UHOH)	0.01M CaCl2	5.12	15.07	-	-	Loamy sand	0.76
91	Weinberg	Germany	UHOHb [01b]	Long-term unfertilized low-P grassland soil	2013	LA Chemie (UHOH)	0.01M CaCl2	7.10	30.00	-	-	Loam	2.41
92	Zamberk_A	Czech Republic	CULS [03]	-	2015	LA Chemie (UHOH)	0.01M CaCl2	4.80	80.00	-	-	Silt loam	1.68
93	Zamberk_B	Czech Republic	CULS [03]	-	2016	Local lab	0.01M CaCl2	4.80	42.10	-	59.30	Silt loam	-
94	Citov	Czech republic	CULS [03]	-	2017	Local lab	0.01M CaCl2	7.70	-	1.10	7.95	Choose from list	-
